# Calredoxin represents a novel type of calcium-dependent sensor-responder connected to redox regulation in the chloroplast

**DOI:** 10.1038/ncomms11847

**Published:** 2016-06-14

**Authors:** Ana Karina Hochmal, Karen Zinzius, Ratana Charoenwattanasatien, Philipp Gäbelein, Risa Mutoh, Hideaki Tanaka, Stefan Schulze, Gai Liu, Martin Scholz, André Nordhues, Jan Niklas Offenborn, Dimitris Petroutsos, Giovanni Finazzi, Christian Fufezan, Kaiyao Huang, Genji Kurisu, Michael Hippler

**Affiliations:** 1 Institute of Plant Biology and Biotechnology, University of Münster, 48143 Münster, Germany; 2 Institute for Protein Research, Osaka University, Suita Osaka 565-0871, Japan; 3 Core Research for Evolutional Science and Technology (CREST), Japan Science and Technology Agency (JST), Saitama 332-0012, Japan; 4 Key Laboratory of Algal Biology, Institute of Hydrobiology, Chinese Academy of Sciences, Wuhan, Hubei 430072, China; 5 Centre National Recherche Scientifique, Unité Mixte Recherche 5168, Laboratoire Physiologie Cellulaire et Végétale, F-38054 Grenoble, France; 6 Commissariat à l'Energie Atomique et Energies Alternatives, l'Institut de Recherches en Technologies et Sciences pour le Vivant, F-38054 Grenoble, France; 7 Université Grenoble 1, F-38041 Grenoble, France; 8 Institut National Recherche Agronomique, UMR1200, F-38054 Grenoble, France

## Abstract

Calcium (Ca^2+^) and redox signalling play important roles in acclimation processes from archaea to eukaryotic organisms. Herein we characterized a unique protein from *Chlamydomonas reinhardtii* that has the competence to integrate Ca^2+^- and redox-related signalling. This protein, designated as calredoxin (CRX), combines four Ca^2+^-binding EF-hands and a thioredoxin (TRX) domain. A crystal structure of CRX, at 1.6 Å resolution, revealed an unusual calmodulin-fold of the Ca^2+^-binding EF-hands, which is functionally linked via an inter-domain communication path with the enzymatically active TRX domain. CRX is chloroplast-localized and interacted with a chloroplast 2-Cys peroxiredoxin (PRX1). Ca^2+^-binding to CRX is critical for its TRX activity and for efficient binding and reduction of PRX1. Thereby, CRX represents a new class of Ca^2+^-dependent ‘sensor-responder' proteins. Genetically engineered *Chlamydomonas* strains with strongly diminished amounts of CRX revealed altered photosynthetic electron transfer and were affected in oxidative stress response underpinning a function of CRX in stress acclimation.

## Abstract (web summary)

Calcium and redox signalling have important roles in acclimation processes. Here, the authors characterise a protein from *Chlamydomonas reinhardtii* that can integrate calcium and redox-related signalling.

## Graphical abstract

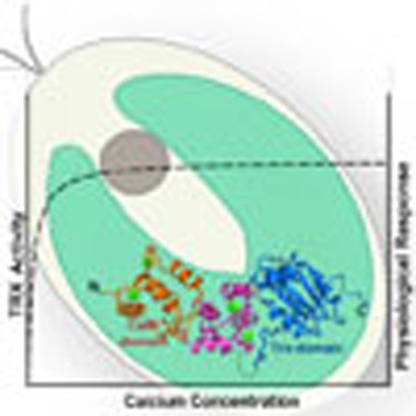

Living organisms respond to dynamic alterations in their environment by matching acclimation strategies to cope with impending stresses. For appropriate cellular responses, cells measure changes in the environment by signal perception and decode signals via signal-transduction pathways to tune their physiology accordingly. In this signal-transduction leitmotif, calcium (Ca^2+^) and redox signalling play important roles in all types of cells from archaea to eukaryotic organisms. For sensing and promotion of Ca^2+^ derived signals, Ca^2+^ sensor proteins are crucial and mostly characterized by the presence of one or more EF-hand Ca^2+^-binding motifs. This motif is highly conserved and EF-hand proteins are encoded in all eukaryotic genomes^1^. Notably, ∼250 EF-hand proteins are encoded in the Arabidopsis genome, more than in any other organism investigated to date^2^.

In line, Ca^2+^-dependent responses are central in acclimation of plants towards environmental changes. Numerous environmental cues cause fluctuations in the cytosolic-free Ca^2+^ concentration, fundamental in the initiation of the appropriate physiological response of the plant (for review ref. 3). Two types of signalling components decode changes in cellular Ca^2+^ concentration in plants. Type I ‘sensor-responder' proteins possess both Ca^2+^-binding and enzymatic ‘effector' domains. Type II components, such as calmodulin (CaM), have a Ca^2+^-binding domain but do not exhibit an enzymatic activity and are designated as ‘sensor-relay' proteins. CaM are small proteins and possess a pair of Ca^2+^-binding EF-hand motifs^4^. Interestingly, in plants the CaM family is extended by a large number of CaM-like proteins (CMLs), which differ from canonical CaMs by variations in length and/or by their number of EF-hands^5^. The physiological role of CaMs and CMLs in plants has been described thoroughly (for recent reviews see refs 5, 6). Although apparent CaM-dependent processes have also been described for chloroplasts (as reviewed in refs 7, 8), no chloroplast-localized CaMs and CMLs have been identified so far.

Alhough, there is increasing evidence that chloroplasts are part of the cellular Ca^2+^ network and contribute to the cytosolic Ca^2+^ signalling, thereby modulating the action of downstream cytosolic ‘sensor-responder' and ‘sensor-relay' proteins. Ca^2+^ is taken up into the chloroplast in the light but released into the cytosol in the dark (as reviewed in ref. 8). The chloroplast-localized Ca^2+^ sensor protein (CAS) may contribute to cellular Ca^2+^ signalling. CAS function is crucial for stomatal regulation^9^,^10^ and chloroplast-mediated activation of immune signalling^11^ in *A. thaliana*. Moreover, it is involved in photo-acclimation in *C. reinhardtii* by contributing to the control of expression of LHCSR3 (ref. 12). LHCSR3 is crucial for qE (ref. 13), the energy-dependent component of non-photochemical quenching, required for efficient light to heat dissipation. Effective qE requires the acidification of the thylakoid lumen and is therefore functionally connected to photosynthetic cyclic electron flow (CEF), which contributes to the pH-gradient across the thylakoid membrane^14^. In microalgae and vascular plants, CEF operates via an NAD(P)H dehydrogenase (NDH)-dependent and/or PROTONGRADIENT REGULATION5 (PGR5)-related pathway^15^. The thylakoid protein PGR5-Like 1 (PGRL1) participates in the PGR5-dependent CEF pathway^16^,^17^,^18^ and associates together with PGR5 with photosystem I (PSI), thereby facilitating the operation of CEF (ref. 18). Recent data further indicated that PGRL1 might operate as a ferredoxin-plastoquinone reductase^19^. Activity of CEF is modulated via the availability of Ca^2+^ (ref. 20). Moreover, there is evidence that CEF is redox controlled^21^ in *C. reinhardtii* and activated by hydrogen peroxide in Arabidopsis^22^. The redox control could operate via the chloroplast thioredoxin (TRX) system and its redox regulation of the PGR5 and PGRL1 cycle^19^, required for PGRL1 homodimer to monomer conversion. In line, reversible activation/inactivation of CEF has been described to operate with an apparent midpoint potential of -306 mV, consistent with a TRX-mediated redox modulation of a thiol/disulfide couple of PGRL1 (ref. 23).

TRXs are protein oxidoreductases that harbour a redox-active dithiol/disulfide motif in their active site. In its reduced state this motif allows cleavage of a disulfide bond of a target protein via a bimolecular nucleophilic substitution reaction. Oxidized TRXs are reduced by TRX reductases (TRXRs). In contrast to other organisms, plants and cyanobacteria possess two independent TRXRs, a ferredoxin-dependent (FTR) and a NADPH-dependent (NTR) and various types of TRXs^24^. NTRC, which resides in the chloroplast, is present in cyanobacteria and plants and has a TRX sequence fused to the C-terminal sequence of NTR^25^. TRXs play a central role in redox regulation and are interlinked with signal-transduction pathways in acclimation responses^26^. The TRX system is in particular important for chloroplasts and the regulation of photosynthesis^24^ as evidenced by, for example, its involvement in metabolic regulation and anti-oxidative stress response. Here, the reduction of oxidized 2-Cys peroxiredoxins (PRXs), which catalytically reduce hydrogen peroxide and organic peroxides, by the TRX system, such as ACHT1 (ref. 27) and NTRC^28^, provides a strong link of the latter to antioxidant metabolism^29^. This is exemplified by the fact that PRXs also operate as redox sensors^30^, thereby further strengthening the link between ROS and redox signalling networks.

Herein we provide evidence that Ca^2+^- and redox-dependent regulation is directly linked within one protein, involved in the regulation of photosynthetic redox regulation and oxidative stress defense. This protein is designated as calredoxin (CRX, Cre03.g202950.t1.1) and consists of 4 EF-hands that are functionally connected to a TRX domain. In this work we characterized functional properties of CRX, which is conserved in the green algae lineage^31^ but absent in vascular plants.

## Results

### Recombinant CRX displays Ca^2+^-dependent TRX activity

**Figure 1:**
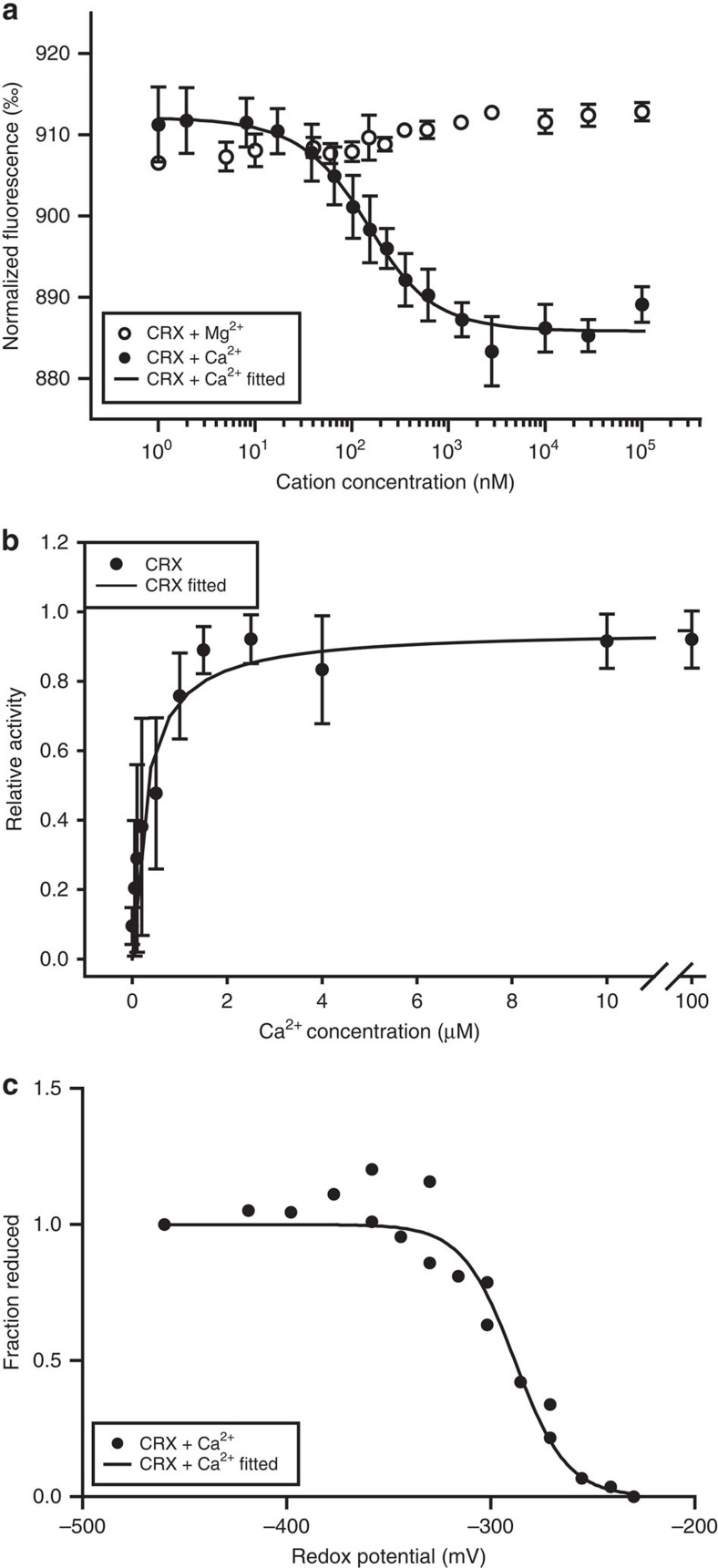
*In vitro* characterization of calredoxin. (**a**) *K*_d_ estimation for Ca^2+^-binding by MST measurements. Fluorescently labelled recombinant WT CRX (CRX) was incubated with defined concentrations of free Ca^2+^ (filled circles) or Mg^2+^ (open circles) and the ratio of detected fluorescence before and after the thermophoretic movement was plotted against the corresponding cation concentration. Data for CRX incubated with Ca^2+^ were fitted according to the law of mass action (black line) and gave a *K*_d_ of 88.2 nM±16.5 nM. Each data point represents the mean value of at least three experiments (±s.d.). (**b**) CRX shows Ca^2+^-dependent redox activity. 10 μM recombinant WT CRX (closed circles) was reduced by *E. coli* NTR and NADPH in defined Ca^2+^ concentrations for 10 min at RT. 200 μM DTNB were added as substrate for reduction by CRX and the increase in absorption at 412 nm was recorded to calculate the redox activity (slope 0–80 s after addition of DTNB). Data were normalized on the highest activity measured for each protein purification and fitted by Michaelis–Menten kinetics (*K*_d_: 281.1±153.8 nM). Error bars represent s.d. of three independent measurements. Assay modified after ref. 62. (**c**) Oxidation–reduction titration of WT CRX. The disulfide/dithiol redox state at each *E*_h_ value was monitored using the monobromobimane fluorescence method. The line represents a fit of the data to a two-electron Nernst curve and yielded an *E*_h_ of −288.2±5.3 mV. Data were acquired in two independent experiments.

Recombinant CRX was isolated and purified via Ni^2+^-NTA affinity chromatography. Binding of divalent cations to recombinant CRX was measured using Microscale Thermophoresis in the presence of calcium and magnesium. For calculation of Ca^2+^ and Mg^2+^ dissociation constants, the ratio between the fluorescence of the fluorescently labelled protein before and after the thermophoretic movement was determined at increasing cation concentrations (Fig. 1a). An increase in Mg^2+^ concentration did not change the fluorescence ratio. On the other hand, an increase in Ca^2+^ concentration led to a clear decrease in the fluorescence ratio, indicating specific binding of Ca^2+^ but not Mg^2+^ to CRX. The Ca^2+^ response data were fitted, resulting in a dissociation constant (*K*_d_ value) of 88.2±16.5 nM. To assess the activity of the TRX domain, an assay was employed in which CRX was reduced by NTR and NADPH, and its activity was measured photometrically via reduction of 5,5′-dithiobis-(2-nitrobenzoic acid) (DTNB). To determine whether the TRX activity of CRX depends on the presence of calcium, the activity assay was performed at increasing concentrations of Ca^2+^ (Fig. 1b). As shown, the TRX domain of CRX is active and its full activity requires calcium. Fitting the TRX- and Ca^2+^-dependent activity curve by Michaelis–Menten kinetics revealed a half maximum activity at 281.1±153.8 nM free Ca^2+^. Importantly, a site-directed mutant of CRX where both cysteine residues of the TRX active site were altered to Ser (C238S, C241S) exhibited only minor DTNB reduction activity, proving that an active TRX domain is required for CRX-dependent substrate reduction (Supplementary Fig. 1a). Notably, the overall Ca^2+^ dissociation constant as measured by Microscale Thermophoresis (88 nM) is slightly smaller to the value obtained from the TRX activity assay (281 nM), suggesting that for TRX activity all four EF-hands are occupied by Ca^2+^. Next, we determined the redox midpoint potential of the TRX active domain by measuring the disulfide/dithiol redox state using the monobromobimane fluorescence technique^32^. The resulting redox titration curve was fitted by the Nernst equation (Fig. 1c). The best fit is represented by a two-electron Nernst curve (see solid line, Fig. 1c) and yields a redox midpoint potential of −288.2±5.3 mV, which is close to the redox midpoint potential of chloroplast TRX *f* from spinach and pea^32^.

### CRX is localized in the chloroplast

**Figure 2:**
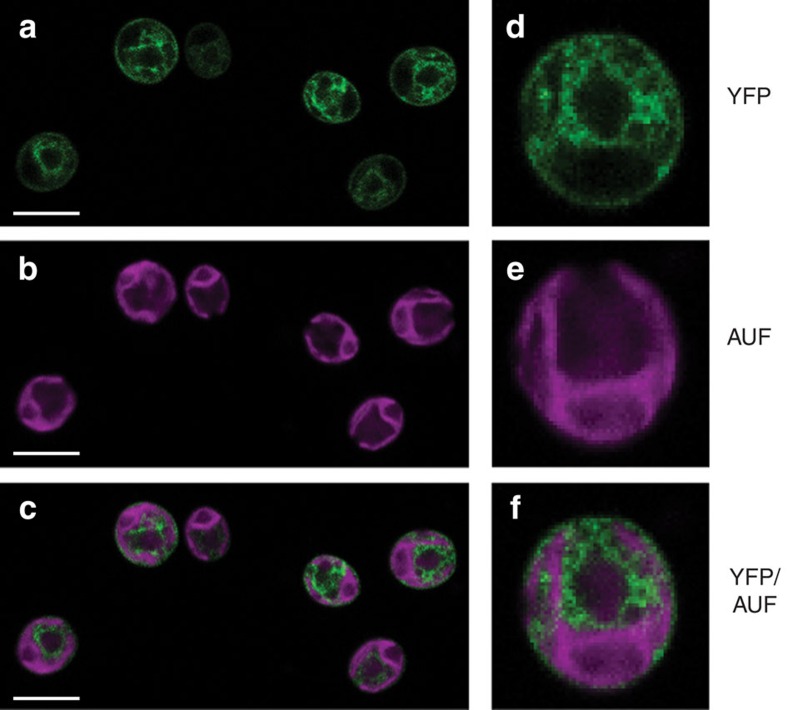
Chloroplast localization of calredoxin in *C. reinhardtii.* Microscopy images of a transgenic strain expressing *ble-2A-calredoxin-mVenus*. (**a**,**d**) YFP fluorescence (detected with a 525–555 nm filter) and (**b**,**e**) chlorophyll fluorescence (AUF, detected with a 690–740 nm filter). Merged images (YFP/AUF) are shown in the bottom row (**c**,**f**). The second row (**d**–**f**) shows the fluorescence signals in a single cell at higher magnification. Scale bars, 20 μm.

ChloroP^33^ and PredAlgo^34^ protein sequence analyses indicated that the nuclear encoded CRX possesses a transit peptide and is accordingly predicted to reside in the chloroplast. To verify this localization, chloroplasts were isolated and assayed for the presence of CRX. Immunoblot analyses revealed that CRX is enriched in chloroplasts versus whole cells and isolated mitochondria (Supplementary Fig. 2). Independently, localization of CRX was assessed by engineering strains that express a *ble-2A-crx-mVenus* construct, a technique utilized previously for subcellular localization studies in *Chlamydomonas*^35^. Confocal laser-scanning microscopy revealed presence of YFP fluorescence in transformed *Chlamydomonas* cells (Fig. 2a,d). The YFP fluorescence partially overlaps with chlorophyll autofluorescence (Fig. 2b,e) as also visualized in the merged images (Fig. 2c,f). The CRX-YFP fluorescence is nearly identical with recently documented mCherry fluorescence signals stemming from a construct targeted to the chloroplast stroma via the *psad* transit peptide sequence^36^. Therefore, we conclude that CRX is localized in the chloroplast stroma of *Chlamydomonas*. Chloroplast localization of CRX was further independently confirmed by transient expression of a *crx*-mCherry construct in *Nicotiana benthamiana* epidermal leaf cells (Supplementary Fig. 3a–c). Here, the *Chlamydomonas* *crx* transit peptide drives chloroplast localization of CRX-mCherry in tobacco, as shown for other transiently expressed *Chlamydomonas* green fluorescent protein constructs having also their endogenous transit peptide sequences^20^.

### CRX interacts with chloroplast 2-cys peroxiredoxin

**Figure 3:**
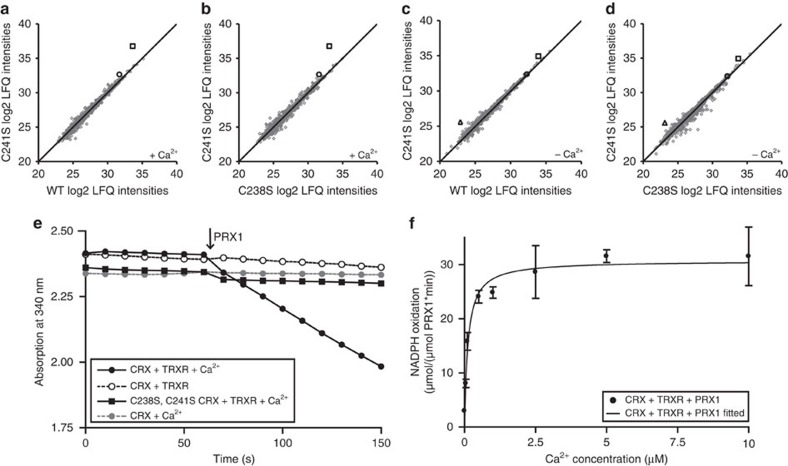
Potential calredoxin interaction partners. (**a**–**d**) Results from the CRX affinity chromatography. WT and mutated versions of recombinant CRX were immobilized on a CNBr-activated resin and a whole-cell lysate of heterotrophically grown *C. reinhardtii* was added to the column to supply potential CRX target proteins. The log2 protein intensities after label-free quantification (LFQ) of the C241S sample were plotted either against the intensities of the WT (**a**,**c**) or the C238S (**b**,**d**) sample. Two proteins were repeatedly (two experiments) significantly more abundant in the C241S sample when Ca^2+^ was present on the column (**a**,**b**): PRX1, Cre06.g257601.t1.2 (open square) and another 2-cys peroxiredoxin, Cre02.g114600.t1.2 (open circle). Elimination of Ca^2+^ (**c**,**d**) reduced the abundance of these proteins and led to identification of a third potential target protein: TRXL1 (Cre03.g157800.t1.1, open triangle). (**e**) Interaction of CRX and PRX1 *in vitro*. 5 μM recombinant CRX was reduced by *E. coli* TRXR and NADPH in the presence of 40 μM H_2_O_2_ at RT. The NADPH absorbance at 340 nm was monitored until a steady decrease was observed. After subsequent addition of 1 μM oxidized recombinant PRX1 (indicated by the arrow) NADPH oxidation was increased in the presence of Ca^2+^ (black filled circle, 44.3 μmol NADPH min^−1^μmol PRX1^−1^) in contrast to CRX without Ca^2+^ (open circle) or without reductase (grey filled circle). CRX C238S, C241S (black filled square) was not able to reduce PRX1. One exemplary measurement is shown. Assay modified after ref. 66. (**f**) Titration of electron transfer between PRX1 and CRX in dependence of Ca^2+^. Fitting the data to Michaelis–Menten kinetics revealed a half-maximal rate of NADPH oxidation at a concentration of 122.3±64.5 nM free Ca^2+^. Scale bars give s.d. of three measurements.

To identify CRX protein–protein interaction partners, we have altered the CRX cysteine residues of the active disulfide to serine and performed thiol-trapping experiments as described before^37^,^38^,^39^. To this end, wild type (WT) and mutated versions of recombinant CRX were immobilized on a CNBr-activated resin and a whole-cell lysate of photoheterotrophically grown *C. reinhardtii* was added to the column to supply potential CRX target proteins to the column. The C241S mutated CRX was most efficient in trapping potential targets due to the missing resolving cysteine in the active site of the TRX domain. The potential targets were eluted with 10 mM dithiothreitol (DTT), digested tryptically and analysed by mass spectrometry. The log2 intensities after label-free protein quantification of the C241S sample were plotted either against the intensities of the WT (Fig. 3a) or the C238S (Fig. 3b) sample. Most proteins were detected at equal ratios (black line represents ratio=1). Two proteins were significantly enriched in the C241S sample: PRX1, Cre06.g257601.t1.2 (open square) and another 2-cys PRX, Cre02.g114600.t1.2 (open circle; see also Supplementary Data 1). In an independent experiment whole-cell lysates stemming from photoautotrophically grown *Chlamydomonas* cells were subjected to the thiol-trapping protocol (Supplementary Fig. 4a–c, Supplementary Data 2). In concurrence, PRX1 was the only candidate significantly higher, abundant in the C241S sample. Notably, PRX1 is a chloroplast-localized 2-cys PRX^39^. Importantly, trapping experiments in the absence of Ca^2+^ revealed particularly diminished enrichment of PRX1, suggesting that binding of CRX to PRX1 requires Ca^2+^ (Fig. 3c,d). Instead, a new protein, a TRX-like protein with unknown function (TRXL1, Cre03.g157800.t1.1) was higher abundant in C241S-dependent enrichment as compared with the two controls (Fig. 3c,d). An activity assay confirmed that CRX is indeed able to reduce PRX1 (Fig. 3e) and drive detoxification of H_2_O_2_ via PRX1 *in vitro*. Moreover, the reduction of PRX1 via CRX is dependent on Ca^2+^ as addition of EGTA diminishes the reduction of PRX1 by CRX about 10-fold (Fig. 3e). A titration of the electron transfer between PRX1 and CRX in dependence of Ca^2+^ revealed a half-maximal rate of NADPH oxidation at a concentration of 122.3 ±64.5 nM free Ca^2+^, a value close to the one found in the Microscale Thermophoresis experiment (Fig. 1a). Notably, the rate of PRX1 reduction via CRX, with a value between 30–44 μmol NADPH min^−1^ μmol^−1^ PRX1 (Fig. 3e,f), is almost as efficient as described for NTRC and PRX in Arabidopsis^28^, thereby supporting a role of CRX in ROS defense as electron donor to PRX1 in chloroplasts of *C. reinhardtii*.

### Crystal structure of CRX

Recombinant CRX was purified from the soluble supernatant with and without adding calcium ions and both were used for crystallization. Although extensive crystallization trials were carried out, only the sample containing calcium could be crystallized. Analytical X-ray fluorescence spectroscopy of the sample without Ca^2+^ revealed that the purified CRX still had small amounts of Ca^2+^ bound even after EGTA treatment and gel-filtration chromatography, thereby causing structural non-homogeneity and hindering crystallization of CRX without Ca^2+^.

Crystals of CRX were obtained at 20 °C. After optimization of the conditions, plate-shaped crystals were grown within 2–3 days using 1.5 M LiSO_4_ and 0.1 M MES-NaOH buffer pH 6 as a precipitant. Crystals directly dipped into liquid nitrogen for the cryo-experiment belonged to the monoclinic space group of *P*2_1_ (Table 1). The maximum resolution of the collected X-ray data set was 1.6 Å. To solve the phase problem, we prepared selenomethionine (SeMet) substituted proteins. The structure was determined using the single-wavelength anomalous dispersion method at 2.8 Å with the SeMet derivative crystal. The phases of the native crystal data were obtained by the molecular replacement method and the structure was refined to 1.6 Å resolution. The crystallographic data and refinement statistics are listed in Table 1(for further details see Supplementary experimental procedures).

**Figure 4:**
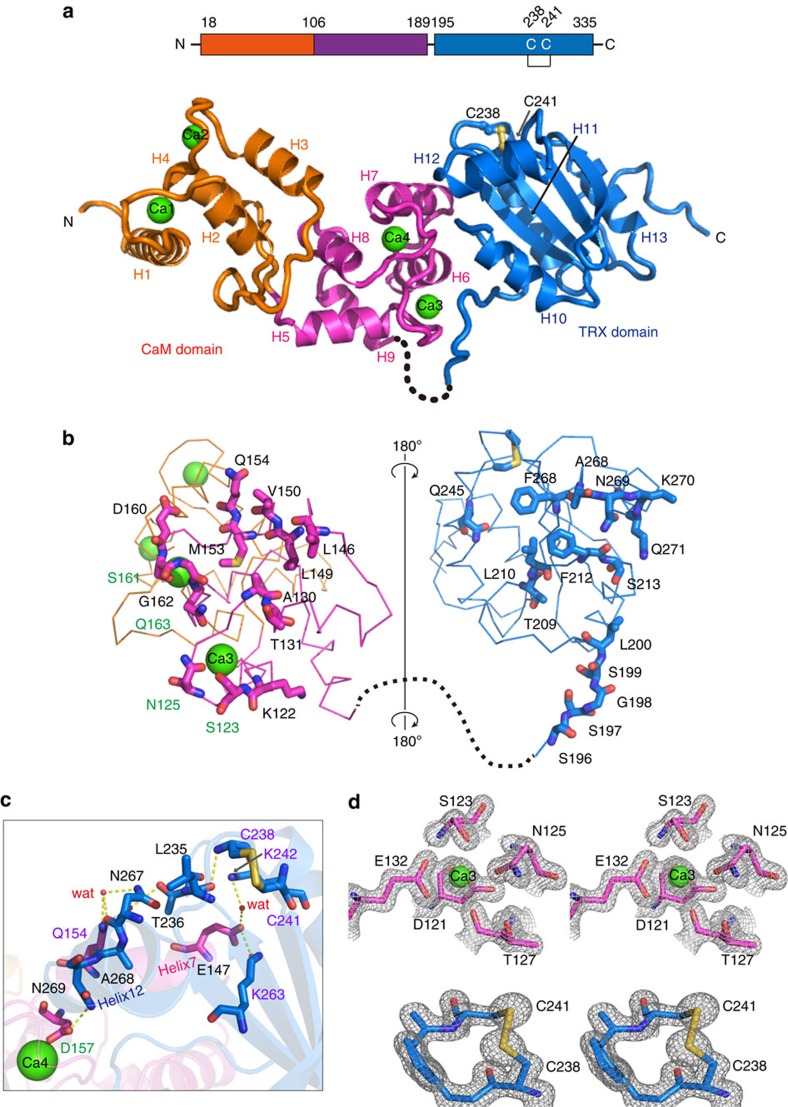
X-ray structure of calredoxin. (**a**) Overall structure of CRX. Sequence diagram of CRX is shown at the top. The N- and C-subdomains of the CaM domain are displayed in orange and magenta, respectively. Bound Ca^2+^ ions are represented as green spheres. The TRX domain is highlighted in marine-blue with the disulfide bridge shown as yellow ball-and-stick model. (**b**) Open-book representation of interactions between the CaM and the TRX domains. Residues involved in direct inter-domain interactions except for water-mediated hydrogen bonds are shown. (**c**) Inter-domain networks between the Ca4 calcium ion in the CaM domain and the disulfide bridge in the TRX domain. Purple labels indicate the residues of structure based mutagenesis. (**d**) The stereo image of 2|F_o_ |-|F_c_ | electron density maps (2*σ* level) showing amino acid residues around Ca3 (upper) and the disulfide bridge between Cys238 and Cys241 (lower).

The crystal contained two CRX molecules per asymmetric unit and the electron density map was clear enough to refine the model without any non-crystallographic restraint. Molecules A and B contained 313 and 310 amino acid residues, respectively, except for flexible regions, four calcium ions in each CRX molecule and 1,156 water molecules in total. Since the model of molecule A contained more structural information than that of molecule B and those were almost identical (r.m.s.d=0.71 Å for Cα carbons), we described the structure of monomeric CRX by referring to molecule A unless otherwise mentioned (Fig. 4a).

The CRX molecule consists of two domains connected by a flexible linker region: the CaM domain with four Ca^2+^-binding EF-hand loops and the TRX domain with one disulfide bridge. In the CaM domain, one calcium ion was bound in each EF-hand loop located in the N- and C-subdomains, respectively. There were nine α-helices (from H1 to H9) in the CaM domain, two of which sandwiched each EF-hand motif. An additional structured loop was inserted between the helices H2 and H3 and the last helix, H9, was connected to the flexible linker region. The TRX domain exhibited the typical TRX fold, possessing four α-helices (H10–H13) and one mixed β-sheet. Side chains of Cys238 and Cys241 formed the disulfide bridge between the second β-strand and the H11 helix. We have added 1 mM DTT in the crystallization droplets, nevertheless the resultant structure contained the disulfide bridge, likely due to the oxidation of DTT during crystallization and to the negative redox midpoint potential of CRX.

At the interface of the two domains, extensive inter-domain interactions were found (Fig. 4b,c). Eighteen residues from the CaM domain and 21 from the TRX domain were involved in the direct inter-domain interaction. Among them, Ser123 and Asn125 provided ligands to the third calcium ion (Ca3; Fig. 4b,d) and Ser161 and Gln163 to the fourth (Ca4; Fig. 4b). Additionally, four residues (Thr127, Glu132, Glu147 and Asp157) from the CaM domain and six residues (Glu215, Glu216, Thr236, Lys242, Lys263 and Asn267) from the TRX domain mediated the inter-domain hydrogen bond network through water molecules (partly shown in Fig. 4b,c). The inter-domain networks between the Ca4 calcium ion in the CaM domain and the disulfide bridge in the TRX domain is shown in Fig. 4c. Strikingly, when the TRX domain is expressed without the CaM domain, purified from *Escherichia coli* and functionally analysed, the protein is not active, neither in the DTNB assay nor in the PRX1 reduction assay (Supplementary Fig. 1a). To validate the importance of the inter-domain hydrogen bond network, residues Gln154, Lys242 and Lys263 were modified by site-directed mutagenesis and altered recombinant CRX proteins were expressed and measured for their competence to donate electrons to PRX1 (Fig. 4c, purple labels indicate the residues of structure based mutagenesis, Supplementary Fig. 1b). CRX mutants Gln154Ala and Lys242Leu were less efficient in electron transfer to PRX1 at low Ca^2+^-concentrations as compared with WT CRX but had similar maximal electron transfer rates. Mutant CRX Lys263Ile on the other hand had an almost threefold lower maximal electron transfer rate at saturating Ca^2+^-concentrations. These results indicate that the communication between Ca^2+^-binding and TRX domains indeed tunes the Ca^2+^-dependent enzymatic activity of CRX.

### CRX depletion leads to increased CEF and ROS production

**Figure 5:**
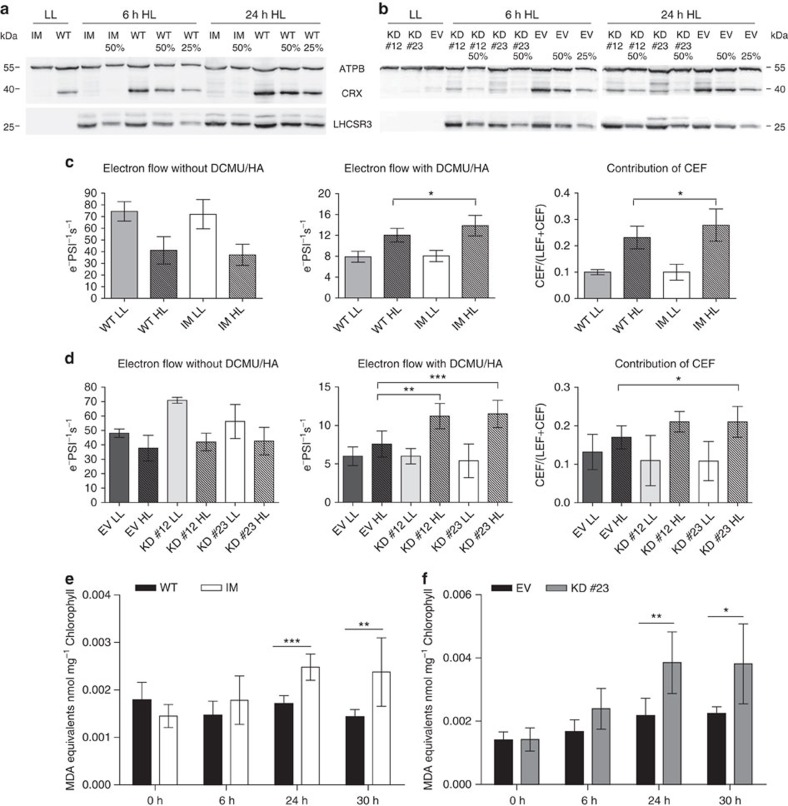
Calredoxin function *in vivo*. (**a**,**b**) Immunoblot analysis of WT versus IM*crx* (**a**) or empty vector (EV) versus *amiRNA-crx-12/23* (KD#12/KD#23) (**b**) whole-cell extracts. ATPB, LHCSR3 and CRX protein expression were examined in cells grown under photoheterotrophic (TAP) LL (30 μE m^−2^ s^−1^), which were shifted to photoautotrophic (HSM) HL (180 μE m^−2^ s^−1^) growth conditions. Chlorophyll (1.5 μg) were loaded per lane and equate 100%. ATPB was used as loading control. (**c**,**d**) Comparison of linear photosynthetic electron flow and CEF in WT versus IM*crx* (**c**) or EV versus KD#12 and KD#23 (**d**) cells grown under TAP/LL, which were shifted for 6 h either to HSM/LL or HSM/HL growth conditions. LL and HL data (±s.d.) refer to analyses of three biological replicates for WT and IM*crx*, with 5 and 11 as well as 6 and 12 measurements, respectively; KD LL Data (±s.d.) refer to analyses of three biological replicates for EV, KD#12 and KD#23 with three measurements each; HL Data (±s.d.) refer to analyses of three (EV), three (KD#12) and seven (KD#23) biological replicates, with three, nine and 10 measurements, respectively. For statistical analysis of indicated data *t*-test with *P<0.05, **P<0.01 and ***P<0.001 was performed. (**e**,**f**) Measurement of lipid peroxidation in calredoxin-deficient strains under HL conditions. WT versus IM*crx* (**e**) and of empty vector (EV) versus KD#23 (**f**) cells after transition from TAP/LL to HSM/HL growth conditions. Malondialdehyde equivalents were measured from whole cells (4 μg Chl ml^−1^) before (0 h, grown in TAP at 30 μE m^−2^ s^−1^) and after HL treatment (shifted to HSM for 6, 24, 30 h at 180 μE m^−2^ s^−1^). WT versus IM*crx* data refer to four biological replicates with eight measurements each, EV versus KD#23 data refer to analyses of three biological replicates with six measurements each. Error bars represent s.d.'s. Statistical comparison of indicated data was done using *t*-test with *P<0.05, **P<0.01 and ***P<0.001.

**Figure 6:**
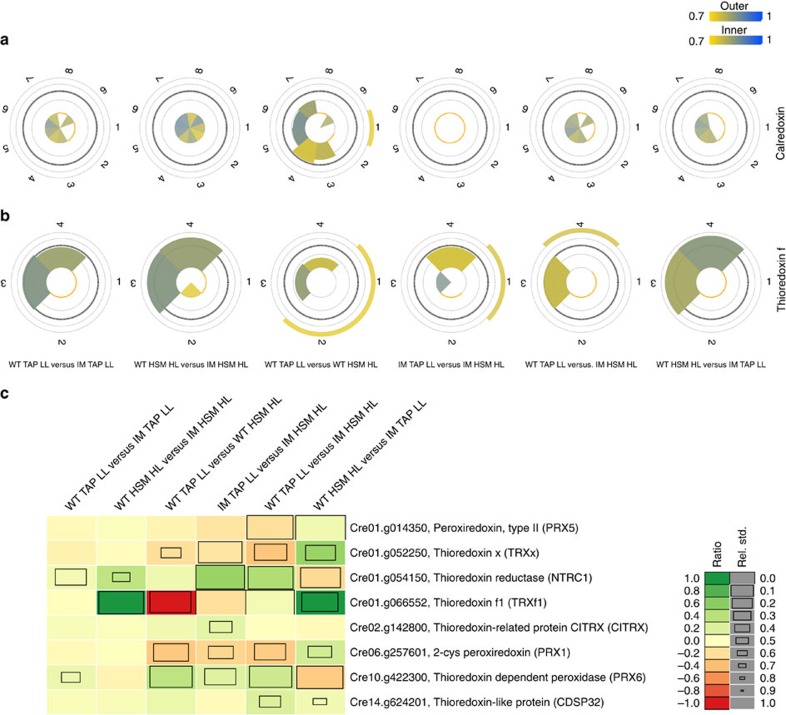
TRX *f* is diminished in HL on depletion of CRX. MS-based ^15^N metabolic labelling-based quantitation from CRX (Cre03.g202950.t1.1) (**a**) and TRX *f* (Cre01.g066552.t1.1) (**b**). Each pie slice represents a quantified peptide. The area of a pie slice is proportional to the log2 ratio of the corresponding peptide between the indicated conditions. Colours represent pyQms quantification score; 0.7 (yellow, false discovery rate (FDR)≤1%) to 1 (blue, prefect match). (**c**) Selected knowledge based TRX community. The heat map represents the ratios of the proteins ranges from yellow to green indicating an upregulation and from yellow to red indicating a downregulation. The s.d. is visualized by the size of the box, smaller the box higher the s.d. of the protein ratio (see legend). For quantitative MS analysis, ^14^N-/^15^N-labelled WT and ^15^N-/^14^N-labelled IM*crx* were mixed based on equal protein amount (total protein amount 100 μg). Eight different conditions were examined as followed: (1) ^14^N-labelled WT TAP LL versus ^15^N-labelled IM TAP LL, (2) ^15^N-labelled WT TAP LL versus ^14^N-labelled IM TAP LL, (3) ^14^N-labelled WT HSM HL versus ^15^N-labelled IM HSM HL, (4) ^15^N-labelled WT HSM HL versus ^14^N-labelled IM HSM HL, (5) ^14^N-labelled WT TAP LL versus ^15^N-labelled WT HSM HL, (6) ^14^N-labelled IM TAP LL versus ^15^N-labelled IM HSM HL, (7) ^14^N-labelled WT TAP LL versus ^15^N-labelled IM HSM HL and (8) ^14^N-labelled IM TAP LL versus ^15^N-labelled WT HSM HL.

To analyse the physiological role of CRX, its expression was diminished using an amiRNA strategy. Moreover, screening of an insertional *Chlamydomonas* mutant library identified a CRX mutant with an insertion in the second intron of the *crx* gene (Supplementary Fig. 5). In the insertional *crx* mutant (IM*crx*) only very minor CRX expression is detectable (IM*crx*, Fig. 5a). Transformants expressing the amiRNA construct (knock down (KD) strains) showed a strong decrease in CRX protein amounts in comparison to the empty vector control (Fig. 5b). Here, *amiRNA-crx-23* and *amiRNA-crx-12* displayed a more than fourfold reduction of CRX expression. Notably, expression of CRX in the WT and the vector control strain is more than fourfold induced in high light (HL) and photoautotrophic conditions as compared with low light (LL) and photoheterotrophic conditions (Fig. 5a,b). This is in line with previous quantitative proteomics results, where CRX expression was found to be induced under autotrophic versus photoheterotrophic conditions^31^ and further confirmed by quantitative mass spectrometric data in this study (Fig. 6a).

To test whether the light-stress response is active in CRX depletion strains, the expression of LHCSR3 was assayed by immunoblotting (Fig. 5a,b). In the IM*crx* as well as in the *amiRNA-crx* strains, expression of LHCSR3 was induced on shift from LL to HL. After 24 h HL, LHCSR3 is slightly diminished in the IM*crx* as compared with the control, while LHCSR3 expression in the KD strains and the vector control strain appears to be similar. Thus the induction of LHCSR3 expression due to an increase in light intensity is operating when CRX amounts are diminished in expression. Measurements of linear photosynthetic electron flow revealed no significant differences between control, IM*crx* and KD strains (Fig. 5c,d). However, measurements of CEF clearly showed a significant increase in CEF capacity in the IM*crx* and the KD strains in comparison to the respective controls (Fig. 5c,d).

Since PRX1 was identified as a potent interaction partner of CRX1, we investigated whether IM*crx* and *amiRNA-crx-23* were affected in ROS defense. Photo-oxidative light stress is known to induce lipid peroxidation in plants^40^. To study lipid peroxidation we employed the thiobarbituric acid assay^41^. Notably, we found that lipid peroxidation is enhanced in both CRX deficient strains (Fig. 5e,f), underpinning a function of CRX in ROS defense and indicating that the functional protein–protein interaction between CRX and PRX1 in detoxification of H_2_O_2_ found *in vitro* (Fig. 3e,f) is of physiological importance *in vivo* (Fig. 5e,f).

### Depletion of CRX diminishes HL induction of TRX *f*

To this end WT and IM*crx* cells were grown either under photoheterotrophic (tris-acetate-phosphate (TAP)) LL conditions (30 μE m^−2^ s^−1^) and shifted to autotrophic (high salt medium (HSM)) HL growth conditions for 6 h (HL, 180 μE m^−2^ s^−1^) in ^15^N or ^14^N labelled media. Mixed whole-cell samples were digested with trypsin using FASP^42^ technology and peptides were analysed by liquid chromatography–mass spectrometry (LC–MS)/MS. We processed samples from eight conditions including swapping experiments (Fig. 6). The mass spectrometric analyses resulted in the identification of 42,815 distinct peptides (pep≤0.05) and 8,555 proteins permitting quantitation and calculation of 2,251 proteins with ratios. No CRX peptides could be identified in IM*crx* underpinning the strong depletion of CRX in IM*crx* (Fig. 6a). These data also revealed that CRX was twofold upregulated after 6 h HL HSM in the WT, in accordance to the immunoblot results (Fig. 5a,b). Quantitative analyses of photosynthetic proteins displayed only slight differences between WT and IM*crx* under HL HSM. Herein core proteins of PSI and PSII were slightly diminished in IM*crx*, an impact that was not observed under LL TAP growth conditions (Supplementary Fig. 6). Co-regulation analyses of the quantitative data were performed by using pyGCluster^43^ including 1,289 proteins present in all clustered conditions. After 250.000 iterations, 146 clusters were found (for a threshold for 0.001) at a minimal cluster length of 4 (Supplementary Fig. 7). These data indicated that overall quantitative responses found via clustering are very similar between the WT and IM*crx*. For example, HL responses including proteins involved in carbon concentrating mechanism (cluster 113, Supplementary Fig. 7) were likewise induced. A striking difference in the HL response was observed for TRX *f* (Cre01.g066552.t1.1), a protein not seen in the clusters. TRX *f* is a chloroplast TRX with crucial functions in the activation of the Calvin–Benson–Bassham (CBB) cycle enzymes in *C. reinhardtii*^44^. As for CRX, TRX *f* was found to be twofold induced under HL in the WT (Fig. 6b). However, the protein was not increased in abundance in IM*crx* under HL HSM in comparison to LL TAP control, an expression difference was also seen in its downregulation under IM*crx* HSM HL versus WT HSM HL (Fig. 6b). This behaviour is very particular for TRX *f*, as it was not seen for other quantified chloroplast TRX proteins (Fig. 6c). Notably, PRX1 was only marginally induced under HL and was not differentially expressed between WT and IM*crx*.

## Discussion

In this work we characterized a protein designated as CRX, representing a new class of Ca^2+^-dependent ‘sensor-responder' proteins. Our structural data revealed that all EF-hands of CRX have the competence to bind Ca^2+^ at high affinity (Figs 1, 3 and 4) and are functionally interconnected with the TRX domain that is enzymatically active (Figs 1 and 3e,f).

A closer look at the interface of the two domains in the CRX structure provided deep insight into the inter-domain communication path. There were two hydrogen bond networks between the active site of the TRX domain and the Ca4 binding site. One network involved Helix 7, a part of the fourth EF-hand motif that was linked to Lys242 located underneath the disulfide bridge (Fig. 4c). The other was through the Helix 12 of the TRX domain. Asn269 located in the end of Helix 12 was directly interacting with the calcium ligand, Asp157 and its side chain represents an integral part of a tight hydrogen bond network stretching along Gln154, Leu235, Thr236, Asn267, Ala268 and Asn269 to the amide nitrogen of Cys238. This latter network included van der Waals interactions and hydrogen bonds with and without water molecules. These two inter-domain pathways are likely responsible for the Ca^2+^-dependent TRX activity (Fig. 1b) and the diminished activity towards PRX1 in the absence of Ca^2+^ (Fig. 3e,f), which is further underpinned by the fact that CRX mutant versions harbouring the alterations Gln154Ala and Lys242Leu were less efficient in electron transfer to PRX1 at low Ca^2+^-concentrations (Supplementary Fig. 1b). The difference in Ca^2+^-dependent TRX activity could also be interpreted in a way that the active refolding of CRX and stimulation of TRX activity and/or binding of NTR required Ca^2+^. However, it appears that the TRX activity of CRX needs Ca^2+^, as the TRX domain without the CaM domain is functionally inactive (Supplementary Fig. 1a). This is an intriguing aspect as the structure of the TRX domain showed noteworthy similarity to the conventional TRX molecule (r.m.s.d=1.36 Å for Cα atoms of spinach TRX *f*), strongly suggesting that the Ca^2+^-dependent activity of CRX is modulated via the inter-domain communication path which is absent in TRX *f*.

Assuming the TRX's interaction sites are conserved in the TRX domain of CRX, the structures of CRX together with the NTR or FTR were predicted based on the published X-ray structures of their electron transfer complex. In the predicted structures (Supplementary Fig. 8a,b), no overlap was found in the CRX:NTR complex; however, in the CRX:FTR, a spatial overlap was found between the FTR and the CaM domain. Based on these *in silico* modellings, it is suggested that CRX is reduced through an NTR-dependent pathway in chloroplasts and oxidized by a 2-Cys PRX from a structural point of view. The effective reduction of CRX via the NTR from *E. coli* is consistent with this interpretation (Figs 1 and 3e).

For the structural analysis of the CRX:PRX complex, we referred to the structure of the TRX domain of ERp46 complexed with the C-terminal peptide of mammalian 2-Cys PRX, PRX4, (TRX:PRX4-peptide)^45^. A mammalian PRX4 existed as a ring-shaped decameric form^46^, while the plant-type PRX forms a noncovalent homodimer^47^. Although it is not clear yet which oligomeric state *Chlamydomonas* PRX1 has, the active site structure is conserved between these PRX structures. From the partial complex structure of TRX:PRX4-peptide, we can build the predicted CRX:PRX-peptide structure (Supplementary Fig. 8c). The TRX domain of CRX has an open space for PRX binding. Additionally, the decameric PRX4 (PDB ID: 3VWU) can be modelled by fitting it's C-terminal peptide to the corresponding peptide in the CRX:PRX-peptide structure (Supplementary Fig. 8d). Further the modelled structure shows a close fit to the open space formed by CRX that implies an additional interaction between CRX and PRX mediated by the CaM-like domain on binding of Ca^2+^. The structural arrangement of the four EF-hands within the Ca^2+^-binding domain of CRX is different with regard to the conventional CaM structure. Referring to the dumbbell-shaped CaM structure, two α-helices (H4 and H5) connecting the second and the third EF-hand motifs correspond to the central helix that would bend when recognizing the target protein. The position of the target protein in the conventional structure of the CaM:peptide complex is occluded by the additional structured loop between H2 and H3 helices, which suggested no similarity in target recognition by CRX. Clearly, target recognition of CRX in dependence of Ca^2+^ via the four EF-hands needs to be further investigated. However, the second protein–protein interaction module of CRX is its TRX domain, as revealed by the capture of PRX1 in the thiol-trapping experiments (Fig. 3a–d, Supplementary Fig. 4a–c). Remarkably, the thiol-trapping of PRX1 as well as CRX electron transfer towards PRX1 were Ca^2+^-dependent (Fig. 3a–f). Thus, as mentioned above, one important function of the Ca^2+^-dependent structural changes of the four EF-hands is manifested in the modulation of TRX binding capability and activity via the inter-domain communication path.

Thereby, it would translate alterations in chloroplast Ca^2+^ concentration and redox potential in modulation of CRX function, placing the protein at the crossroad of redox and Ca^2+^ signalling in the chloroplast. Notably, CRX is the first CML protein identified in the chloroplast. Its presence and function strongly supports the notion that Ca^2+^ plays an important role in regulating algal photosynthesis but also underpins the view of a general functional interconnection of Ca^2+^ and redox signalling in chloroplast photosynthesis.

Consistent with this hypothesis, our *in vivo* analysis reveals that CEF is induced on reducing or removing CRX activity. This effect on CEF can be rationalized based on the more reducing stromal redox poise and/or enhanced ROS (for example, hydrogen peroxide production (see above)) when CRX activity is diminished. The strong depletion of CRX enhanced lipid peroxidation (Fig. 5e,f), in line with an increased oxidative stress. As described above, CRX is an efficient alternative to the NTRC system^28^ to reduce hydrogen peroxide via PRX1 (Fig. 3e,f). Therefore, a strong diminishment of CRX should results in a less efficient reduction of oxidized PRX1, thereby increasing in hydrogen peroxide in the light. Importantly, while CRX as well as PRX1 expression is enhanced at increasing light intensities (Fig. 5a, Fig. 6a and ref. 39), the induction of CRX in HL is much stronger than the increase of PRX1 (Fig. 6c). Besides NTRC as shown in Arabidopsis^28^, TRX*x* is another chloroplast TRX^48^, is involved in electron transfer to PRX1 in *Chlamydomonas*^49^. TRX*x* expression is comparable between WT and IM*crx*, while NTRC expression in WT is not induced in HL but slightly diminished under HL in IM*crx* (Fig. 6c), overall suggesting that in HL, CRX is a significant electron donor to PRX1, probably crucial in minimizing photo-oxidative damage.

The increase in chloroplast Ca^2+^ in the light could be important for activation of the TRX domain of CRX (Figs 1b and 3e). Light and Ca^2+^-dependent activation has also been described for other chloroplast enzymes, for example, some of the CBB cycle (reviewed in ref. 7). In this scenario CRX would provide reducing power for oxidized PRX1 in the light. It is striking that on depletion of CRX the expression of TRX *f* is not induced in HL (Fig. 6b,c). As mentioned, TRX *f* is critical for the activation of CBB cycle enzymes^44^ and is the only chloroplast TRX that is glutathionylated^50^, impairing its reduction by FTR leading to compromised light activation of target enzymes^50^. Regulation of TRX *f* via CRX is therefore another and new layer of regulation, which needs to be further explored. Here the diminishment of NTRC in IM*crx* is also noteworthy, indicating that the depletion of CRX has a particular impact on the chloroplast TRX system.

Notably, the Ca^2+^-dependent reduction of PRX1 via CRX (Fig. 3f) was found at a *K*_d_ value of ∼122 nM free Ca^2+^, a value that corresponds to the resting stromal Ca^2+^-concentration^8^. Thus an increase of stromal Ca^2+^ could indeed further activate CRX and accelerate the rate of PRX1 reduction or reduction of other substrates. Therefore, enhanced ROS in the light due to depletion of CRX is a possible explanation for the induction of CEF in *C. reinhardtii* as described for Arabidopsis^22^. In Arabidopsis, however, hydrogen peroxide induced CEF via the NDH-dependent pathway and not via the PGR5-dependent pathway. It is currently not known whether the NDA2-dependent pathway in *C. reinhardtii*^51^ is activated by hydrogen peroxide. In the redox regulation of the PGR5 and PGRL1 cycle, the PGRL1 homodimer to monomer conversion requires reduction of a disulfide bond via the TRX system^19^. Although CRX is depleted and TRX *f* is not induced in HL in IM*crx*, CEF is still activated and higher than WT. CEF activation could be accomplished by other chloroplast TRXs such as TRX*x*, which is not affected in IM*crx* (Fig. 6c). On the other hand the diminishment of TRX *f* in HL may impair the activation of CCB. This in turn would feedback on the photosynthetic electron transfer, as stromal ATP and NADPH will increase, and activate CEF (refs 52, 53), in line with our findings (Fig. 5c,d). In conclusion, CRX is involved in ROS defense and in controlling TRX *f* expression, thereby modulating the stromal redox poise and concomitantly CEF.

Moreover, the CRX inter-domain communication path could translate alterations in chloroplast Ca^2+^ and redox state into cellular signalling.

## Methods

### Culture conditions

All *C. reinhardtii* strains were grown photoheterotrophically in TAP medium^54^ at 25 °C, 120 r.p.m. shaking and 30 μE m^−2^ s^−1^. For HL growth experiments, cells were shifted to photoautotrophic growth conditions in HSM, washed once with HSM, resuspended to a chlorophyll concentration of 4 μg ml^−1^ and exposed to HL (180 μE m^−2^ s^−1^) for indicated hours.

### Protein expression and purification

For *in vitro* experiments *E. coli* strain BL21 (DE3; Novagen) was transformed with pET-22b(+) plasmids encoding different versions of CRX (WT; C238S; C241S; C238S, C241S; Q154A; K242L; K263I or the TRX domain only). For protein crystallization, CRX WT and the SeMet derivative were expressed from the same vector but in *E. coli* Rosetta and B834 (DE3) pLysS strain, respectively. Cells were grown at 37 °C until an OD_600_ of 0.6 was reached. Expression was induced with 0.5 mM isopropyl-β-D-thiogalactoside at 37 °C for 5 h (*in vitro* experiments) or with 0.1 mM Isopropyl-β-D-thiogalactopyranoside at 20 °C for 18–24 h (crystallization experiments). The His-tagged proteins were purified either natively from *E. coli* lysates (for crystallization, microscale thermophoresis (MST) and PRX1 interaction experiments) or under denaturing conditions from inclusion bodies (DTNB activity assay (except Supplementary Fig. 1), redox titration, thiol-trapping experiment) using Ni^2+^-affinity resin. Eluates were dialyzed against 30 mM MOPS, 100 mM KCl, pH 7.2 and sterile filtered. For protein crystallization after Ni^2+^-affinity purification the protein sample was submitted to ion exchange (Hi-Trap Q) chromatography. The eluates were pooled and further purified by gel filtration (Superdex-200).

### Crystallization and structure solution

WT CRX was crystallized at 20 °C using the hanging-drop vapour diffusion method. Protein concentration was adjusted to 10 mg ml^−1^. Sample buffer contained 20 mM Tris-HCl pH 8.0, 150 mM NaCl, 1 mM DTT and 150 mM CaCl_2_. Precipitant solution was 1.5 M LiSO_4_ dissolved in 0.1 M MES-NaOH buffer pH 6. To obtain the crystals, the drops contained 1 μl of the protein and 1 μl of precipitant solution. SeMet derivative crystals were grown with the same experimental setting. X-ray diffraction images were collected at 100 K using a synchrotron radiation source with 0.90000 Å for native and 0.97500 Å for SeMet. All data sets were collected at the SPring-8 using the BL44XU beamline.

The native and SeMet derivative data sets were processed using the HKL-2000 software. Phase problem was solved by the single-wavelength anomalous dispersion method using Phenix AutoSol in Phenix program suite at 2.8 Å resolution. The SeMet derivative protein had 32 SeMet residues and 27 sites were found. The density modified resultant electron density map was clear enough for interpretation and the asymmetric unit contained two molecules of CRX. The model was build using COOT software along with the NCS-averaged electron density map calculated using DM in the CCP4 package. Crystallographic refinement was performed using REFMAC5. The final structure was validated using wwPDB validation server. The analysis showed that all residues were in the favoured region of Ramachandran plot, with no residues as outliers. Crystallographic data and refinement statistics are shown in Table 1.

### Vector constructions

RNA isolation from *Chlamydomonas reinhardtii* was performed using TRI reagent solution (Sigma Aldrich) and 300 ng of DNase I treated RNA was used for cDNA synthesis (iScript cDNA Synthesis Kit, Biorad) according to the manufacturer's protocol.

For *in vitro* experiments the *crx* and *prx1* genes were amplified from cDNA lacking the transit peptide and the stop codon using the primers 5′-ggaattccatatgtgcagcgctcgctcc-3′ and 5′-cgggatcctgcagggccgccagc-3′ for CRX and 5′-ggtattccatatggcttcccacgccgag-3′ and 5′-cgggatcctgcacggcagagaagtactcc-3′ for PRX1. Each amplicon was digested with *Nde*I and *Bam*HI (NEB) and cloned into the pET-22b(+) vector (Novagen). Based on the CRX plasmid, site-directed mutagenesis (QuickChange II Site-Directed Mutagenesis Kit, Agilent) was performed (C238S; C241S;C238S, C241S; Q154A; K242L and K263I) according to the manufacturer's protocol. The following primers were used for the mutagenesis reaction: C238S: 5′-gcgcgctcacatggagccggccctgcaaggg-3′ and 5′-cccttgcagggccggctccatgtgagcgcgcagc-3′; C241S: 5′-ggtgccggcccagcaagggcatg-3′ and 5′-catgcccttgctgggccggcacc-3′; C238S, C241S: 5′-gctcacatggagccggcccagcaagggcat-3′ and 5′-atgcccttgctgggccggctccatgtgagc-3′; Q154A: 5′-gtcgtccttgtcgtacatggccatgaggtcaaccagcttc-3′ and 5′-gaagctggttgacctcatggccatgtacgacaaggacgac-3′; K242L: 5′-ccgctgcatgcccaggcagggccggca-3′ and 5′-tgccggccctgcctgggcatgcagcgg-3′; K263I: 5′-gttggcgttaccgaacagaatcacaaacacgatgtggtc-3′ and 5′-gaccacatcgtgtttgtgattctgttcggtaacgccaac-3′. Moreover the same plasmid was used as template for the amplification of the TRX domain of CRX with the primers 5′-ggtataccatatgctggacgaggcggg-3′ and 5′-ccgctcgagcaggttggcgatcag-3′. The amplicon was digested with NdeI and XhoI (NEB) and cloned into an empty pET-22b(+) vector (Novagen).

To visualize calredoxin expression in *C. reinhardtii in vivo* a calredoxin-YFP construct was generated. For this purpose the *crx* gene without the stop codon was amplified using the primers 5′-ctaggctagcatgattgcaattcgcac-3′ and 5′-ctagtccggacagggccgccagc-3′. Then the amplicon was sequentially digested with *Nhe*I and *Bspe*I (NEB) and cloned into a pChlamiRNA3-based vector, between the *bleomycin-FMDV 2A* sequence^35^ and the YFP coding region^55^. Nuclear transformation of *C. reinhardtii* strain CC-851 cw2 mt+ was performed using the glass bead protocol (1–2 μg of plasmid DNA per transformation, linearized with *Kpn*I and *Not*I)^56^. Transformants were selected on TAP plates supplemented with zeocin (10 μg ml^−1^).

Calredoxin-knockdown strains were generated using an amiRNA approach^57^. Calredoxin-specific oligonucleotides were designed using the WMD3 tool (http://wmd3.weigelworld.org/cgi-bin/webapp.cgi). The oligonucleotides 5′-ctagtTCGGATCAGTTTTGTTGTTCAtctcgctgatcggcaccatgggggtggtggtgatcagcgctaTGAAGAACAAAACTGATCCGAg-3′ and 5′-ctagcTCGGATCAGTTTTGTTCTTCAtagcgctgatcaccaccacccccatggtgccgatcagcgagaTGAACAACAAAACTGATCCGAa-3′ that target the 3′UTR of the *crx* gene were annealed and ligated via the *SpeI* restriction site into the pChlamiRNA2 vector. The resulting plasmid was transformed into the arginine auxotrophic cw15-325 (cwd mt+ arg7 nit1+ nit2+) strain by electroporation according to ref. 58, with some modifications. Plasmid DNA (150–300 ng) was added to 250 μl of a concentrated cell suspension (2 × 10^8^ cells ml^−1^ in TAP supplemented with 40 mM sucrose). The cell/DNA mix was incubated in an electroporation cuvette for 10 min on ice before electroporation with pulse settings of 1 kV and 25 μF (GENE PULSER II coupled to the PULSE CONTROLLER II, Biorad). Cells were transferred into a 50-ml falcon tube containing 10 ml TAP supplemented with 40 mM sucrose and shaken overnight under LL (10 μE m^−2^ s^−1^). Cells were collected by centrifugation at 3,000*g* for 5 min and resuspended in 1 ml TAP. Resuspended cells (330 μl) were plated onto 1% (w/v) TAP agar plates.

For the transient expression of the calredoxin-mCherry fusion protein in *N. benthamiana* epidermal leaf cells the *crx* gene without the stop codon was amplified using the primers 5′-ggggtaccatgattgcaattcgcactg-3′ and 5′-catctcgagcagggccgccagc-3′. The amplicon was digested with *Kpn*I and *Xho*I (NEB) and cloned into the binary vector pGPTKII.bar_mcherry (kindly provided by Jörg Kudla).

### Insertional mutagenesis and IM*crx* identification

The IM*crx* mutant was identified from an insertion mutant library using a PCR-based screening method^59^. The library was generated using the AphVIII fragment from plasmid pMJ013b as the insert, and a rescued mutant of ift46-1 (CC-4375) as the parental strain^60^,^61^. The flagella less mutant ift46-1 was rescued by transformation of a ∼7.2 kb fragment including the full-length genomic sequence of IFT46 fused to the YFT gene at 3′-end, and the Aph7′ gene as the selective marker. The whole library was separated into ∼100 super-libraries each containing 1,440 transformants. PCR reactions using super-library DNA as template were performed with a gene specific primer and an insert specific primer LGR06-F (primers are listed in Supplementary Table 1). The super-library that included the IM*crx* mutant was determined according to the sequencing data of a positive PCR product (Supplementary Fig. S5. The positive super-library was further separated into 15 small-libraries and each small-library DNA was checked individually by PCR with appropriate *crx* target primer. The single clone of the IM*crx* mutant was picked from the small library after identification by colony PCR. Furthermore, insertion of the AphVIII fragment was confirmed with a primer pair binding adjacently to the insertion site (EX2-F, EX3-R, Supplementary Table 1, Supplementary Fig. 5).

### Microscale thermophoresis binding analyses

Recombinant CRX (100 μl of 40 μM) was labelled with red fluorescent amine-reactive dye according to the instructions of the MO-L001 Monolith Protein Labeling Kit RED-NHS (amine reactive). A constant concentration of labelled CRX (∼115 nM) was mixed with defined concentrations of free Ca^2+^ or Mg^2+^ ions (up to 100 μM) in 30 mM MOPS (pH 7.2), 100 mM KCl, 0.05% Tween-20, 5 mM total EGTA or EDTA respectively. To prepare solutions with Ca^2+^ in the micromolar range we took advantage of available calcium buffer kits guidelines (ThermoScientific). Measurements were performed at 22 °C in standard treated glass capillaries (Monolith NT Capillaries, NanoTemper) and the thermophoresis analysis was performed on a NanoTemper Monolith NT.115 instrument (20% LED; 20% infrared-laser power). The MST data were fitted with the law of mass action using the NanoTemper Analysis software to obtain *K*_d_ values for binding between CRX and Ca^2+^.

### Measurement of Ca^2+^-dependent redox activity

The redox activity of CRX was determined photometrically in a TRXR-dependent assay^62^ (modified). *E. coli* (40 nM) TRXR were used to specifically reduce CRX (10 μM) by 10 min incubation in 30 mM MOPS, 100 mM KCl, pH 7.2 with NADPH (200 μM) at RT. After addition of 200 μM DTNB, the formation of TNB^−^ was followed at 412 nm (Ultrospec 3000, Amersham Biosciences). The reduction rate of DNTB was determined at defined free Ca^2+^ and Mg^2+^ concentrations and over a time course of 0–80 s after addition of DTNB. In Fig. 1b the different activities were normalized on the highest activity measured for each batch of purified CRX, plotted against the free ion concentration and modelled according to Michaelis–Menten kinetics with GraphPad Prism 2.01 software.

### Determination of redox potential

Oxidation–reduction titration was performed as follows. Briefly, recombinant WT CRX (150 μg ml^−1^) was incubated for 2 h at room temperature in 100 mM MOPS, 2.5 μM CaCl_2_, pH 7.0 that contained a total DTT concentration of 2 mM. Different redox potentials (*E*_h_) were adjusted by mixing appropriate quantities of oxidized and reduced DTT. The CRX disulfide/dithiol redox state at each *E*_h_ value was monitored using the monobromobimane fluorescence method: 250 mM monobromobimane (final concentration) was allowed to react with reduced CRX thiols for 20 min at RT. To remove excess dye, proteins were precipitated with 10% TCA in acetone and washed with 1% TCA in acetone. Pellets were resuspended in 100 mM Tris/HCl pH 8, 1% SDS and fluorescence of a 3:50 dilution in Tris/HCl, pH 8 was detected in a Jasco Fluorometer at 470 nm (excitation: 380 nm). Data were fitted with a two-electron Nernst curve using GraphPad Prism 2.01 software to calculate the CRX *E*_m_ value.

### Transient expression in *Nicotiana benthamiana*

*Agrobacterium tumefaciens* (GV3101 strain), containing the calredoxin-mCherry fusion construct (OD_600_ 0.5), were coinfiltrated with the p19 strain (OD_600_ 0.3) into 6-week-old *N. benthamiana* leaves as described previously^63^. Microscopic analyses were conducted 3 days after infiltration on leaf discs (Ø 1.2 cm) of the lower epidermis at room temperature with water as imaging medium.

### Microscopy

Protein fluorescence was detected by confocal laser-scanning microscopy using a Leica TCS-SP5 II setup consisting of an inverted confocal laser-scanning microscope DMI6000 (Leica) equipped with an × 63/1.2 water immersion lens (HCX PL APO lambda blue 63.0 × 1.20 Water UV). The following filters were used: mVenus, excitation 514 nm (Argon laser), scanning 525–550 nm; mCherry, excitation 561 nm (DPSS Laser), scanning 605–638 nm. Chlorophyll autofluorescence was detected at 690–740 nm (Supplementary Table 2). Image acquisition was performed using the Leica software (Leica Application Suite – Advanced Fluorescence 2.6.0.7266; Leica Microsystems). Images are shown in RGB mode and brightness and contrast were adjusted using Adobe Photoshop CS3 software.

### Calredoxin affinity chromatography

A thiol-trapping approach was performed based on refs 37, 64. Five milligram of either WT CRX or two single-point mutated versions (C238S and C241S) were covalently linked to HCl-washed CNBr-activated sepharose 4B overnight at 4 °C to produce three separate columns. After successful coupling, remaining active groups were blocked with Tris/HCl, pH 8. Columns were equilibrated overnight in binding buffer (50 mM MOPS, 50 mM NaCl and pH 8) either with 2.5 μM free Ca^2+^ in 5 mM EGTA or with EGTA only. Five milligram of total protein from *C. reinhardtii* were loaded onto each column and incubated for 1 h at 4 °C. After washing with 3 × 5 ml 50 mM MOPS, 500 mM NaCl, pH 8.3, potential CRX targets were eluted with 10 mM DTT in 1 ml steps until absorbance at 280 nm dropped significantly, as determined by NanoDrop measurement. The eluates were concentrated in Vivaspin 4 Columns (MWCO 10 kDa, Sartorius) and subsequently submitted to tryptic digestion (Filter Aided Sample Preparation)^42^ for subsequent mass spectrometric analysis.

### Peptide identification by mass spectrometry

Tryptically digested peptides were chromatographically separated using an Ultimate 3000 RSLCnano System (Thermo Scientific). The sample was loaded on a trap column (C18 PepMap 100, 300 μM × 5 mm, 5-μm particle size, 100-Å pore size; Thermo Scientific) and desalted for 5 min using 0.05% (v/v) TFA/2% (v/v) acetontrile in ultrapure water at a flow rate of 5 μl min^−1^. Then the trap column was switched in-line with the separation column (Acclaim PepMap100 C18, 75 μm × 15 cm, 2-μm particle size, 100-Å pore size; Thermo Scientific). The mobile phases for peptide elution consisted of 0.1% (v/v) formic acid in ultrapure water (A) and 80% acetonitrile/0.08% formic acid in ultrapure water (B). Peptides were eluted at a flow rate of 300 nl min^−1^ and employing the following gradient profile: 2.5–35% B over 90 min, 35–60% B over 20 min, 60–99% B over 5 min and 99% B for 10 min. Afterwards the column was re-equilibrated with 97.5% A for 25 min.

The LC system was coupled via a nanospray source to a Q Exactive Plus mass spectrometer (Thermo Scientific) operating in positive ion mode. MS data were acquired in a data-dependent manner, dynamically choosing the 12 most abundant precursor ions from the survey scans (scan range *m/z* 400–1,600, resolution 70,000, AGC target value 1e6, maximum injection time 50 ms) for fragmentation (MS/MS) by higher-energy C-trap dissociation (27% normalized collision energy). Dynamic exclusion was enabled with an exclusion duration of 60 s. AGC target value for MS/MS was 5e4 at 50 ms maximum injection time. The precursor isolation window was 2.0 *m/z* and the resolution for higher-energy C-trap dissociation spectra was set to 17,500. The ‘underfill ratio', specifying the minimum percentage of the target ion value to be reached at the maximum fill time, was 1%. Singly charged ions, ions with charge state 8 and above as well as ions with unassigned charge states were excluded from fragmentation. Internal lock mass calibration on *m/z* 445.12003 was enabled.

### Label-free protein quantification

Peptide identification and protein quantification was performed using MaxQuant 1.5.1.2 (ref. 65) with default parameters for tryptic digestion and Orbitrap detection (maximum peptide mass: 8,000 Da). Spectra were searched against a database containing protein sequences of the *Chlamydomonas* v5.5 gene models (Joint Genome Institute) merged with mitochondrial and chloroplast protein sequences from NCBI databases BK000554.2 and NC_001638.1. The database was further supplemented with commonly observed contaminants (http://www.thegpm.org/crap/). Cysteine carbamidomethylation was set as a fixed modification, the oxidation of methionine, protein N-terminal acetylation, and phosphorylation of serine, threonine and tyrosine were set as variable modifications. A false discovery rate of 1% was applied to peptide and protein identifications. False discovery rate calculation was performed by MaxQuant using a target-decoy approach. The ‘match between runs' setting was activated.

Correlation plots were produced using log2 transformed label-free quantification protein intensities.

### PRX1 interaction assay

The interaction of CRX and PRX1 was measured *in vitro* in a TRXR coupled photometrical assay^66^. Recombinant CRX (5 μM) was reduced by incubation with 1 μM *E. coli* TRXR and 120 μM NADPH in 30 mM MOPS, 100 mM KCl, pH 7.2 and defined free amounts of Ca^2+^ as well as 40 μM H_2_O_2_. The NADPH consumption was measured at 340 nm until a steady decrease in absorption was detected. Then the reaction was started by addition of 1 μM recombinant PRX1 and recording of the absorption at 340 nm was continued. The rate of NADPH oxidation was calculated from the first 60 s after addition of PRX1.

### Immunoblot analysis

Immunoblot analyses were performed using equal amounts of chlorophyll (2 μg, Fig. 5) or the equal amounts of total protein (30 μg, Supplementary Fig. S1)^31^. All primary antibodies were used as follows: CRX 1:3000 (generated by Eurogentec using recombinant CRX for immunization of rabbits), ATPB 1:5,000 (Agrisera), CA 1:1000 (Agrisera), LHCSR3 1:2,000^67^. The signals were detected by enhanced chemical luminescence. An exemplary full-blot containing molecular weight markers is shown in Supplementary Fig. 9.

### Spectroscopic measurements

P700 absorption and electrochromic shift signal measurements were done using an LED pump-probe JTS-10 spectrophotometer (BioLogic). Single turnover measurements were performed using a dye laser emitting at 640 nm, pumped by the second harmonic of a Minilite II Nd:YAG laser (Continuum). *C. reinhardtii* cells were harvested and resuspended to a chlorophyll concentration of 20 μg ml^−1^ in 20 mM HEPES (pH 7.2) containing 10% Ficoll (w/v) and incubated 10 min in the dark under constant shaking to avoid anaerobiosis.

Electron flow rates were determined as the product of k_ox_ [P700_red_] as described in ref. 21. Measurements performed in the absence of inhibitors were utilized to calculate linear electron flow, while measurements in the presence of PSII inhibitors 3-(3,4-dichlorophenyl)-1,1-dimethylurea (40 μM) and hydroxylamine (2 mM) were used to determine rates for CEF around PSI. Continuous illumination was provided at 630 nm with an intensity of 130 μE m^−2^ s^−1^. *k*_ox_ was determined as the initial change in the 520–546 nm electrochromic shift signal at the onset of actinic illumination normalized to the electrochromic shift signal recorded 140 μs after application of a saturating, single turnover flash, in the presence of the aforementioned PSII inhibitors. To assess [P700_red_], continuous actinic illumination was applied for 10 s, followed by a 30-ms short pulse of strong actinic light to fully oxidize P700. P700 absorption changes were followed at 705 nm, corrected by subtraction of the signal at 740 nm.

### Lipid peroxidation measurement

Malondialdehyde equivalents were measured as described^41^, with minor modifications. Cells were grown under photoheterotrophic (TAP) LL (30 μE m^−2^ s^−1^) conditions until a density of 15 μg Chl ml^−1^ was reached and then shifted to photoautotrophic (HSM) HL (180 μE m^−2^ s^−1^) growth conditions at a chlorophyll concentration of 4 μg ml^−1^. At designated time points, two 15 ml aliquots of each culture were taken and incubated with butylated hydroxytoluene (final concentration 0.01% w/v) to prevent further lipid peroxidation. Cells were pelleted at 4,000*g* for 10 min at 4 °C and resuspended in 1.8 ml of degassed extraction buffer (3.75% TCA (w/v) 0.05 N HCL) with or without 0.1% (w/v) thiobarbituric acid. Samples were incubated in a boiling water bath for 15 min and cooled down to room temperature on ice. Cell extracts were pelleted at 4,000*g* for 10 min and the absorbance of the supernatant was determined at 440, 532 and 600 nm. Malondialdehyde equivalents were calculated as described 41 using a molar extinction coefficient for the TBA-malondialdehyde complex of 155,000 nmol^−1^ ml^−1^.

### Isolation of chloroplasts and mitochondria

Chloroplasts and mitochondria were isolated as described in ref. 48.

Protein concentration was determined using the Pierce Protein assay kit (Thermo Scientific, Perbio Science GmbH). Bovine serum albumin was used as standard for construction of the calibration curve.

### Isotopic labelling

Isotopic ^14^N and ^15^N labelling was performed with two biological replicates for the WT and the Calredoxin-deficient IM*crx* strain. Cells grown for 6 h under photoheterotrophic (TAP) LL (30 μE m^−2^ s^−1^) conditions were compared with cells grown for 6 h under photoautotrophic (HSM) and HL (180 μE m^−2^ s^−1^) conditions. For quantitative MS analysis, ^14^N-/^15^N-labelled WT and ^15^N-/^14^N-labelled IM*crx* were mixed based on equal protein amount (total protein amount 100 μg). Eight different conditions were examined as followed: (1) ^14^N-labelled WT TAP LL versus ^15^N-labelled IM TAP LL, (2) ^15^N-labelled WT TAP LL versus ^14^N-labelled IM TAP LL, (3) ^14^N-labelled WT HSM HL versus ^15^N-labelled IM HSM HL, (4) ^15^N-labelled WT HSM HL versus ^14^N-labelled IM HSM HL, (5) ^14^N-labelled WT TAP LL versus ^15^N-labelled WT HSM HL, (6) ^14^N-labelled IM TAP LL versus ^15^N-labelled IM HSM HL, (7) ^14^N-labelled WT TAP LL versus ^15^N-labelled IM HSM HL and (8) ^14^N-labelled IM TAP LL versus ^15^N-labelled WT HSM HL. Proteins were tryptically digested overnight on centrifugal ultrafiltration devices (cutoff: 30 kDa) according to the FASP method^42^ with an enzyme-to-protein ratio of 1:100.

### LC–MS/MS analyses of WT IM*crx* strains

Liquid chromatography and MS parameters were essentially the same as described above with the following modifications:

Peptide chromatography was carried out on an Acclaim PepMap100 C18 capillary column (75 μm × 50 cm, 2-μm particle size, 100-Å pore size; Thermo Scientific). The following gradient was applied: 2.5–35% B over 195 min, 35–60% B over 40 min, 60–99% B over 5 min, 99% B for 15 min.

MS data acquisition was performed using a data-dependent Top20 method (MS1: scan range *m*/*z* 375–1600, AGC target value 1e6, maximum injection time 30 ms. MS2 parameters: see above). The precursor isolation window was 1.5 *m/z*. Full scan resolution was set to 70,000 (FWHM). Some samples were additionally analysed with an MS1 resolution of 140,000 (FWHM).

### Identification of peptide spectrum matches and statistical evaluation

MS2 Spectra were analysed using Ursgal^68^. Post processing was performed using percolator (v. 2.08)^69^ and PSMs were filtered by 5% posterior error probability (PEP). Complete analysis pipeline was executed using the Python framework Ursgal (v 0.3.3)^68^ using default profile ‘QExactive+'. Additionally, Methionine oxidation and N-terminal acetylation was included as post translational modification search parameter. Details on parameters can be inspected in the Ursgal log JSONs that have been uploaded with the raw result files. In total, 3,234,459 and 2,687,720 peptide spectrum matches for PEPs≤5% and ≤1%, respectively, were observed. This translates to 42,815 and 35,926 distinct peptides for PEPs≤5% and ≤1%, respectively. Detailed listing of all identified peptides can be found in Supplementary Material 1.

### Metabolically labelled peptide quantification

All peptides (PEP≤5%) were subjected to quantification using pyQms (refs 31, 70) using default parameters. To quantify peptides irrespectively of their identification in a given LC–MS/MS run, retention time alignment was performed as described before^31^,^70^ All quantifications were grouped and processed using piqDB, an internal database structure based on Python, mongoDB and Hadoop. In total, all quantifications resulted in 1,462,589 matched isotope pattern chromatograms (MICs) containing at least one isotope pattern match with a mScore ≥0.7. However, to include a MIC further into the quantitative analysis a total of eight quantifications (MS1 hits) were required with a mScore ≥0.7. Finally, protein quantifications were based on peptide identification with a PEP threshold ≤5% but with a more stringent quantification mScore threshold of 0.8 (for at least one match within the MIC). All circle plots, showing raw quantification results for all peptide (incl. charge) in all conditions were plotted using piqDB. Detailed listing of all quantified peptides can be found in Supplementary Data 3.

### Unsupervised clustering

In total, 1,289 proteins were quantified in all six conditions and those were subjected to unsupervised clustering using pyGCluster^43^. All possible combinations between the distance matrices ‘Euclidean' and ‘Correlation' and the linkages ‘complete', ‘average', ‘weighted', ‘centroid', ‘median' and ‘ward' were clustered during each of the 250,000 iterations. Finally, all agglomerative hierarchical cluster results were combined using pyGCluster's meta clustering approach resulting in 146 frequent (obCoFreq ≥0.1%) clusters forming five communities (Supplementary Fig. 7), namely community 21, 92, 99, 113 and 139.

### Data availability

Coordinates and structure factors have been deposited in the world-wide Protein Data Bank with accession codes of 5E37. Mass spectrometry data has been uploaded to the ProteomeXchange Consortium with the data set identifier PXD003049 for the affinity trap experiment and PXD003759 for the isotopic labelling experiment. All other data that support the findings of this study are available from the corresponding author on reasonable request.

## Additional information

**How to cite this article:** Hochmal, A.K. *et al.* Calredoxin represents a novel type of calcium-dependent sensor-responder connected to redox regulation in the chloroplast. *Nat. Commun.* 7:11847 doi: 10.1038/ncomms11847 (2016).

## Supplementary Material

Supplementary Information Supplementary Figures 1-9, Supplementary Tables 1-2 and Supplementary References.

Supplementary Data 1 Quantification results for CRX affinity chromatography with and without Ca2+

Supplementary Data 2 Quantification results for CRX affinity chromatography with Ca2+ and HSM grown cells.

Supplementary Data 3 Quantified peptides from the isotopic labeling experiment.

## Figures and Tables

**Table 1:** Data collection, phasing and refinement statistics for single-wavelength anomalous dispersion (SeMet) structures

	**SeMet**	**Calredoxin**
*Data collection*		
Space group	*P*2_1_	*P*2_1_
Cell dimensions		
*a*, *b*, *c* (Å)	85.6, 54.8, 89.1	86.0, 55.2, 89.5
*α*, *β*, *γ* (°)	90.0, 101.1, 90.0	90.0, 101.1, 90.0
Wavelength (Å)	0.97500	0.90000
Resolution (Å)	50-2.8 (2.85-2.80)	50-1.6 (1.63-1.60)
*R*_merge_(%)	9.5 (19)	6 (55.8)
*I /σ I*	60.2 (20.2)	34.9 (3.1)
Completeness (%)	99.6 (100)	99.7 (100)
Redundancy	7.5 (7.6)	3.7 (3.6)
		
*Refinement*		
Resolution (Å)		50-1.6
No. reflections		103,272
*R*_work_/*R*_free_ (%)		19.88/23.25
No. of atoms		
Protein		4,862
Ca^2+^		8
Water		849
*B*-factors		
Protein		25.038
Ca^2+^		24.761
Water		35.824
r.m.s. deviations		
Bond lengths (Å)		0.0253
Bond angles (°)		2.2260

Highest resolution shell is shown in parentheses.
