# Supplementary material for: Calredoxin represents a novel type of calcium-dependent sensor-responder connected to redox regulation in the chloroplast
Source: Nat Commun. 2016 Jun 14;7:11847. doi: 10.1038/ncomms11847 (PMC4911631; doi:10.1038/ncomms11847)
Supplement: Supplementary Information — Supplementary Figures 1-9, Supplementary Tables 1-2 and Supplementary References. [file ncomms11847-s1.pdf]

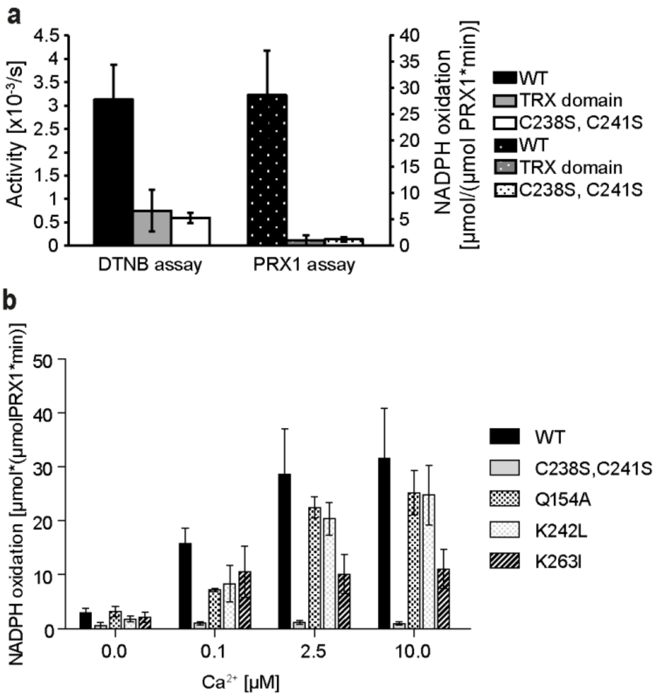

Supplementary Figure 1. Evidence for Ca^{2+} dependent redox activity of CRX mediated by the inter-domain communication path

(a) The activity of natively purified WT (black) and doubly mutated (C238S, C242S; white) CRX as well as the CRX TRX domain only (grey) was measured photometrically either by DTNB reduction at 412 nm (solid columns, left y-axis) or NADPH oxidation in the PRX1 interaction assay at 340 nm (dotted columns, right y-axis) at a free Ca^{2+} concentration of 2.5 µM. Error bars represent standard deviation of three independent measurements. (b) Titration of electron transfer between PRX1 and WT CRX or different CRX mutants (C238S, C242S; Q154A; K242L; K263I) in dependence of Ca^{2+}. 5 µM recombinant CRX was reduced by *E.coli* thioredoxin reductase (TRXR) and NADPH in the presence of 40 µM H_2O_2 for 2 min at RT. The NADPH absorbance at 340 nm was monitored after subsequent addition of 1 µM oxidized recombinant PRX1 and the NADPH oxidation rate was calculated and plotted against the respective Ca^{2+} concentration. Scale bars give standard deviation of three measurements.

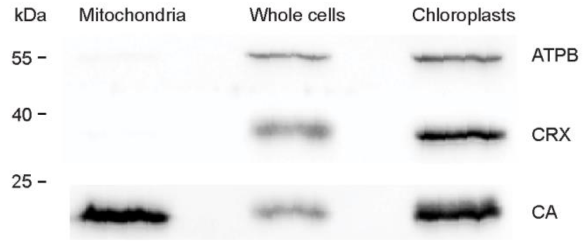

Supplementary Figure 2. CRX is localized in Chlamydomonas chloroplasts

Immunoblot detection of CRX in whole cell extracts, isolated chloroplast and mitochondria fractions of *C. reinhardtii*. Each fraction was loaded with equal amounts of total protein (30 µg). ATPB and mitochondrial carbonic anhydrase (CA) were used as purity and enrichment control of the different fractions.

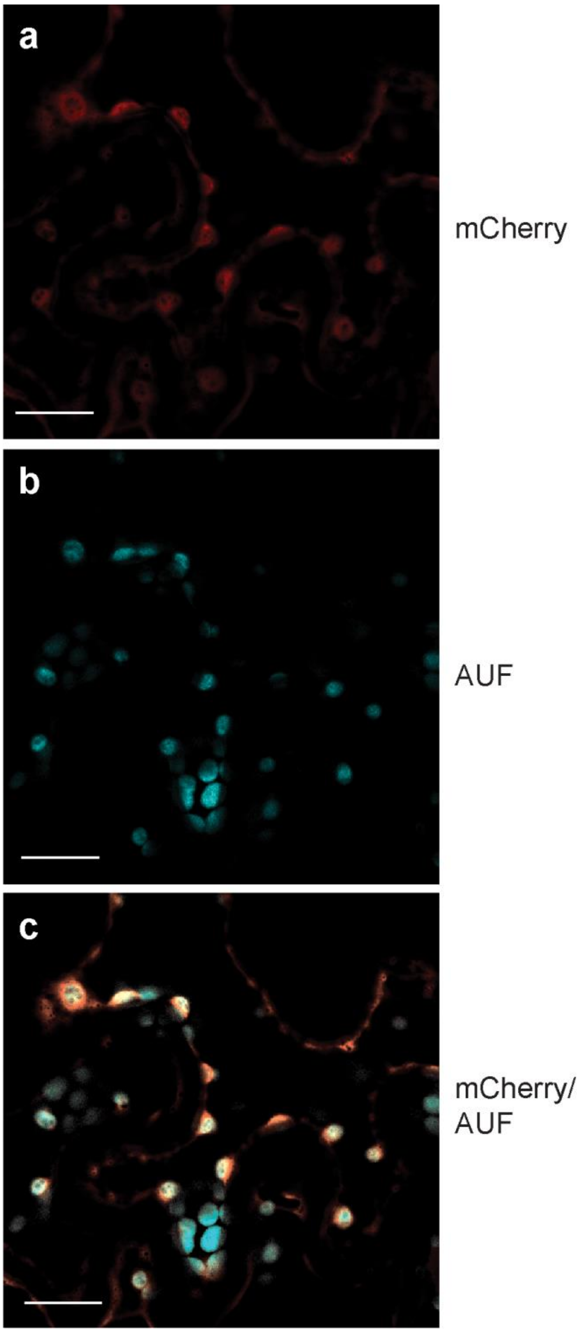

Supplementary Figure 3. Subcellular localization of CRX in tobacco

Transient expression of *crx*-mCherry fusion protein in agro-infiltrated *Nicotiana benthamiana* leaf epidermal cells (3 days post infiltration). Fluorescence of CRX-mCherry (a), autofluorescence (AUF) of the chloroplast (b) and overlay images (c). Scale bars represent 20 µm.

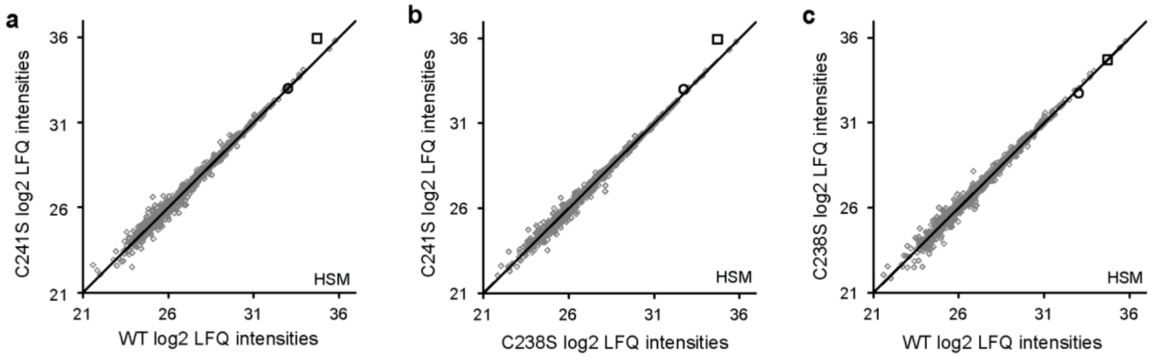

Supplementary Figure 4. PRX1 is a potential CRX interaction partner

(a-c) Results from the second CRX affinity chromatography. WT and mutated versions of recombinant CRX were immobilized on a CNBr-activated resin and a whole cell lysate of *C. reinhardtii* grown in HSM/HL conditions was added in the presence of 2.5 µM free Ca^{2+} to supply potential CRX target proteins to the column. The potential targets were eluted with 10 mM DTT, digested tryptically and analyzed by mass spectrometry. When the log2 protein intensities after label free quantification (LFQ) of the C241S sample were plotted either against the intensities of the WT (a) or the C238S (b) sample, PRX1 (Cre06.g257601.t1.2, open square) was more abundant in the C241S sample. Most other proteins, among them Cre02.g114600.t1.2 (open circle), were detected at equal ratios (black line represents ratio = 1). When the results from the C238S sample were plotted against the intensities of the WT (c) PRX1 is detected at an equal ratio as well.

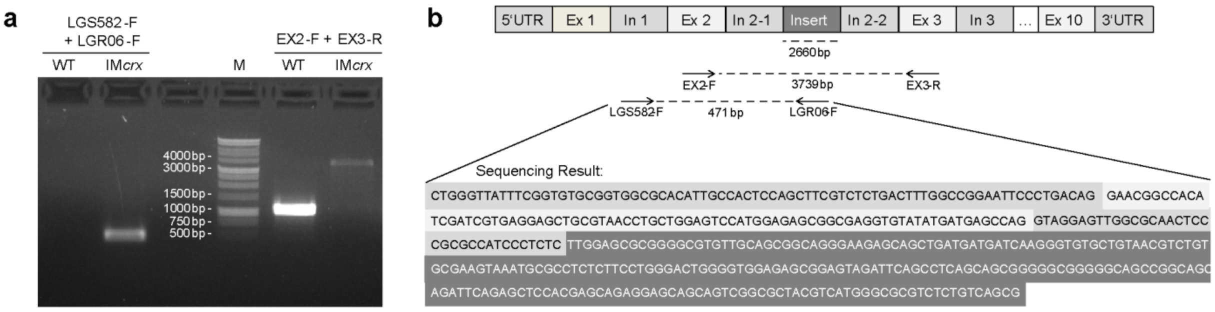

Supplementary Figure 5. Determination of the insertion site for the *crx* insertional mutant Insertion of the AphVIII fragment into the genomic sequence of *crx* was confirmed by a positive PCR product with the screening primer pair LGR06-F and LGS582-F for the genomic DNA of the insertional mutant (a, IM*crx*, lane 2). In contrast, no PCR product was observed for the genomic DNA of the wildtype (a, WT, lane 1). The PCR product for the mutant was purified and sequenced with the primer LGR06-F. As shown in a schematic representation of the genomic sequence (b, Ex: exon, In: intron), the insertion site could be determined as the second intron, which is in agreement with the PCR product size of ca. 470 bp. Furthermore, the use of a primer pair binding adjacently to the insertion site (EX2-F, EX3-R) led to a PCR product with an increased size for IM*crx* (a, lane 6, ca. 3700 bp) in comparison to the WT (a, lane 5, ca. 1100 bp) corresponding to the full length insert sequence. Primer sequences are listed in Supplementary Table 1.

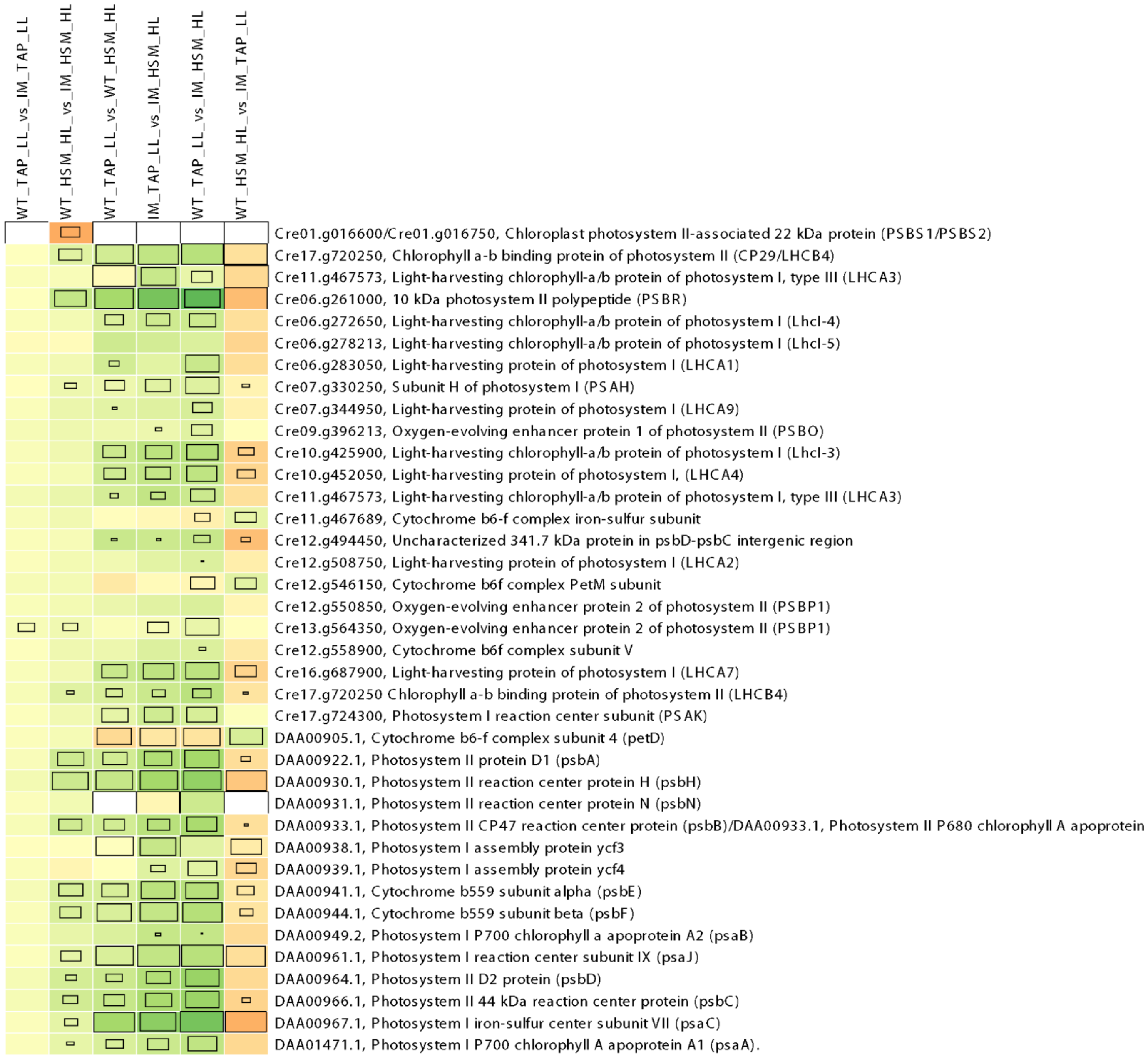

Supplementary Figure 6. Knowledge based heatmaps of photosynthetic proteins. Knowledge based heatmaps of photosynthetic proteins. Each heat map represents the ratios of the proteins ranging from yellow to green to blue indicating an up-regulation and from yellow to orange to red indicating a down-regulation. The standard deviation is represented by the size of the box, the smaller the box, the higher the standard deviation of the protein ratio (see legend). Proteins are annotated manually (short tag) and by their JGI5 Augustus 11 id or by their NCBI protein accession number.

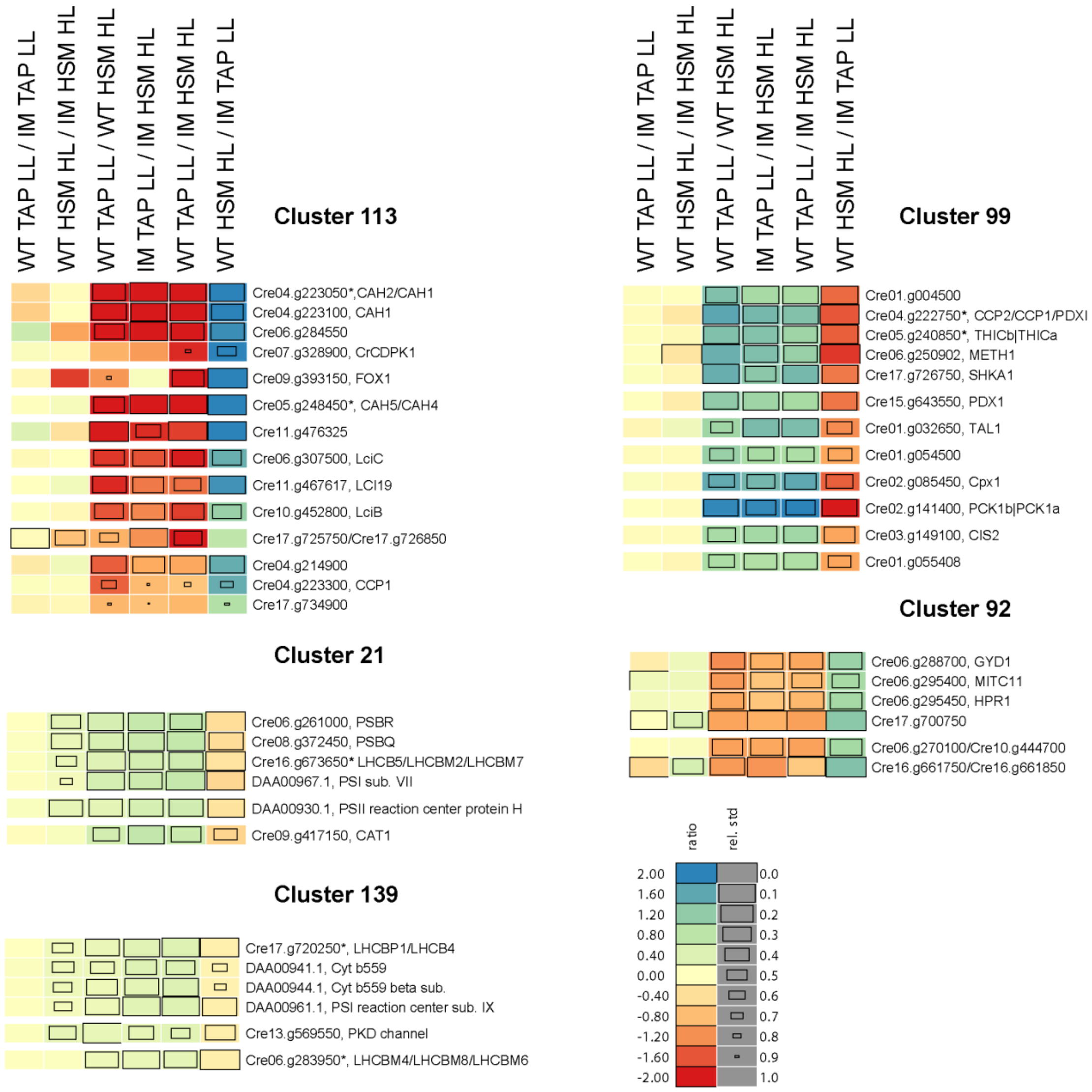

Supplementary Figure 7. Communities obtained by pyGCluster.

Communities obtained by pyGCluster. Communities of the clustered proteins are shown as heat maps and shown as described in Supplementary Fig. 6. Proteins are annotated manually (short tag) and by their JGI5 Augustus 11 id.

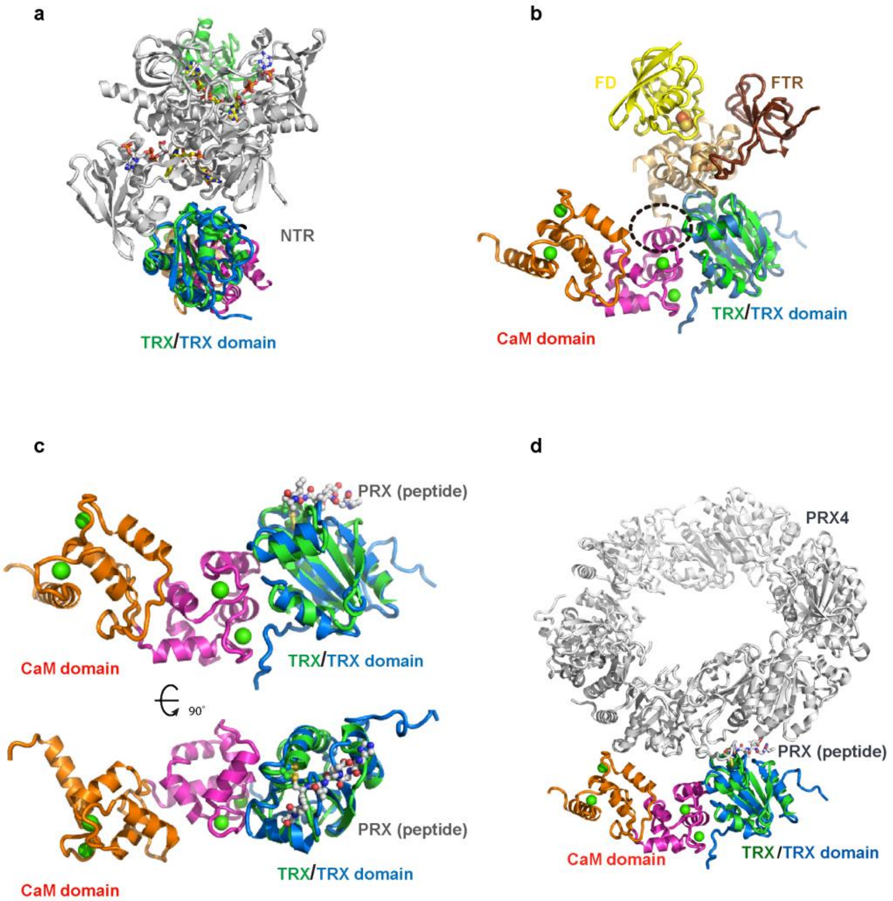

Supplementary Figure 8. Modeling of a CRX-PRX1 electron transfer complex

The electron transfer complex is modeled *in silico* based on the crystal structures of CRX:NTR (PDB ID:1F6M)[1], CRX:FTR (PDB ID:2PVO)[2] and CRX:PRX4 (PDB ID:3WGX)[3,4]. The CaM and TRX domain of CRX are colored in magenta and marine blue respectively. (a) Model of the CRX:NTR complex. Crystal structures of NTR and TRX are colored in grey and light-green. (b) Model of the CRX:FTR:FD complex. Crystal structures of FD, FTR and TRX *f* are colored in yellow, brown and light-green respectively. (c) Model of the CRX:PRX complex. The C-terminal peptides of PRX4 are colored according to each atom color; white carbon, blue nitrogen and red oxygen atoms. The C-terminal peptides of PRX and TRX are shown in ball-and-stick and light-green ribbon models. (d) Predicted model of the decameric mouse PRX4 (PDB ID:3VWU) and CRX based on the complex structure of TRX:PRX-peptide. First, the structure of TRX:PRX-peptide was superimposed on the TRX-domain of CRX. Second, the decameric mouse PRX4 was superimposed based on the peptide region of modeled CRX:PRX-peptide structure.

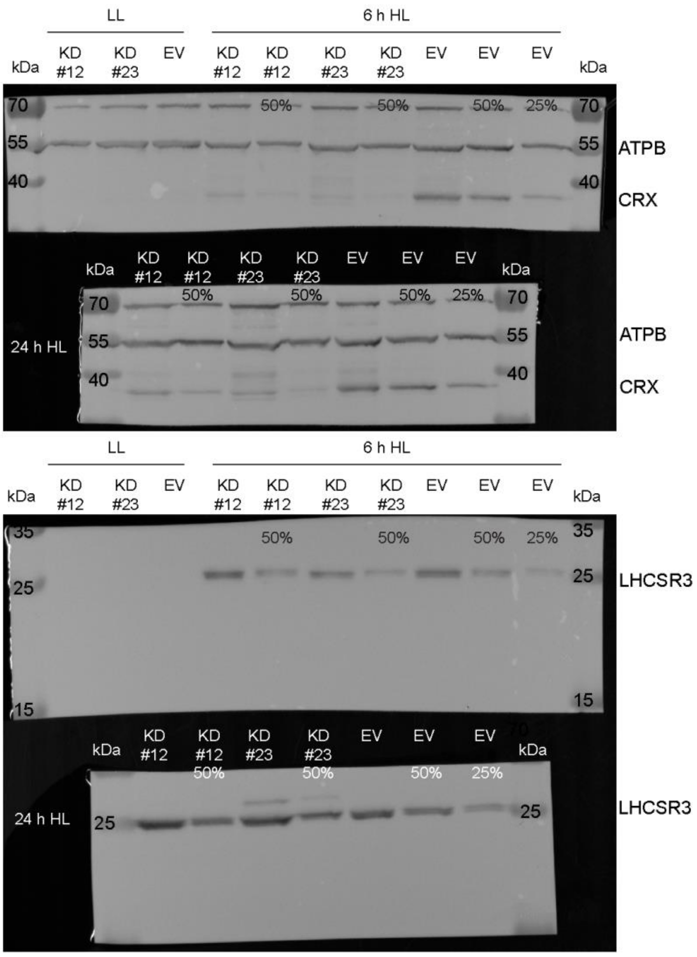

Supplementary Figure 9. Related to Figure 5 Calredoxin function *in vivo*

Immunoblot analysis of or empty vector (EV) vs *amiRNA-crx-12/23* (KD#12/KD#23) whole cell extracts. ATPB, LHCSR3 and CRX protein expression were examined in cells grown under photoheterotrophic (TAP) low light (LL, 30 µE$m^{-2}s^{-1}$), which were shifted to photoautotrophic (HSM) high light (HL, 180 µE$m^{-2}s^{-1}$) growth conditions. 1.5 µg chlorophyll were loaded per lane and equate 100%. ATPB was used as loading control.

Supplementary Tables

Supplementary Table 1. Related to Experimental Procedures (Insertional mutagenesis)

Primer name	Sequence 5´-3´
LGR06-F	GGCGAAGCTTGGTACCGCTA
LGS581-F	GCAGCGTTGTTGGTTCTTCG
LGS582-F	CACAATGCTGGGTTATTTCGG
LGS583-F	GTCTCCCCATTACACCACGAT
LGS584-F	AGTGCGACTGTTCCTTACCGG
LGS585-F	GCTGCGTTTGTAACGAACCT
LGS586-R	CCCGTCCACATCAGCCATCT
LGS587-R	ATCGTGGTGTAATGGGGAGAC
LGS588-R	TGGTTCACCGAGTAAACTATCCC
LGS589-R	CGCCAGTTTCTGTTAGGGTGTT
LGS590-R	ACCGACATTCCATCTCACCC
EX2-F	ACGGCCACATCGATCGTGAG
EX3-R	TTCTCCAGCGACACAGGGTTG

List of primers used for the screening and confirmation of the IM*crx* mutant.

Supplementary Table 2. Equipment and settings used for the confocal microscopy images.

**Confocal Images**	**Scanning range [nm]**	**Gain [HV]**	**Offset [%]**	**Pixel**	**Image Length [µm]**	**Bit**	**AOTF**
Figure 2A	525-555	49.8	-	512 x 512	99.91	8	514 (20%)
Figure 2B	690-740	24.2	-	512 x 512	99.91	8	514 (20%)
Figure S3A	605-638	10	-	512 x 512	108.78	8	561 (20%)
Figure S3B	690-740	873.8	-	512 x 512	108.78	8	561 (20%)

AOTF: acousto-optical tunable filter settings.

Supplementary References

1 Lennon, B. W., Williams, C. H., Jr. & Ludwig, M. L. Twists in catalysis: alternating conformations of Escherichia coli thioredoxin reductase. *Science* **289**, 1190-1194 (2000).

2 Dai, S. *et al.* Structural snapshots along the reaction pathway of ferredoxin-thioredoxin reductase. *Nature* **448**, 92-96, doi:10.1038/nature05937 (2007).

3 Kojima, R. *et al.* Radically different thioredoxin domain arrangement of ERp46, an efficient disulfide bond introducer of the mammalian PDI family. *Structure* **22**, 431-443, doi:10.1016/j.str.2013.12.013 (2014).

4 Echalier, A. *et al.* Crystal structure and solution NMR dynamics of a D (type II) peroxiredoxin glutaredoxin and thioredoxin dependent: a new insight into the peroxiredoxin oligomerism. *Biochemistry* **44**, 1755-1767, doi:10.1021/bi048226s (2005).
